# ¹H NMR Chemical Shifts and J‐Coupling Constants Dependence on Temperature and pH: Implications for the Quantification of Relevant Metabolites

**DOI:** 10.1002/nbm.70239

**Published:** 2026-02-16

**Authors:** Felizitas C. Wermter, Christian Bock, Wolfgang Dreher

**Affiliations:** ^1^ Department of Chemistry, In‐Vivo‐MR Group University Bremen Bremen Germany; ^2^ Integrative Ecophysiology Alfred Wegener Institute Helmholtz Centre for Polar and Marine Research Bremerhaven Germany

**Keywords:** chemical shift, J‐coupling constant, pH, temperature

## Abstract

^1^H MRS enables the non‐invasive measurement of various compounds in biological systems, both in vitro and in vivo. It thus offers the possibility of investigating characteristic metabolic processes and identifying biomarkers under both normal and pathological conditions. Therefore, a reliable quantification of important compounds is essential. Many quantification methods established for this purpose use model spectra based on prior knowledge of chemical shifts and J‐coupling constants. The broad application range of ^1^H MRS also allows its use at different temperatures and pH values based on the physiological conditions of the model organism. Against this background, this work aimed to investigate 15 important metabolic compounds in terms of their temperature and pH dependence of chemical shifts and J‐coupling constants. The results indicate that the majority of the compounds exhibit significant changes in their spectra in response to temperature changes. Additionally, the pH can significantly influence the spectra. Therefore, model functions were calculated to predict the chemical shift and the J‐coupling constants in the investigated range for each combination of temperature and pH. In addition, the influence of incorrect prior knowledge in quantifying the metabolite concentration was analysed. For the higher concentrated metabolites (> 2 mM), only minor errors in the quantification result from the use of incorrect prior knowledge regarding the chemical shifts and the J‐coupling constants, except for Cr and PCr. As the concentration of the metabolite decreases, the percentage error in the estimated concentration increases. Thus, the model functions can be applied to quantifying spectra for various organisms and their specific physiological properties. This optimisation is essential to avoid or minimise errors in quantifying ^1^H MRS data.

## Graphical abstract

^1^H MRS, in combination with quantification methods using model functions based on prior knowledge of chemical shifts and coupling constants, allows the determination of characteristic metabolic processes in healthy and diseased tissue. Using the functional relationships $\delta \left(\mathrm{pH},T\right)$ and $J\left(\mathrm{pH},T\right)$ enables this prior knowledge to be optimised to ensure reliable quantification of spectra even under changing physiological conditions. The functions for the different resonances of a compound (in this case, creatine) often exhibit significant differences in their pH and temperature dependencies.

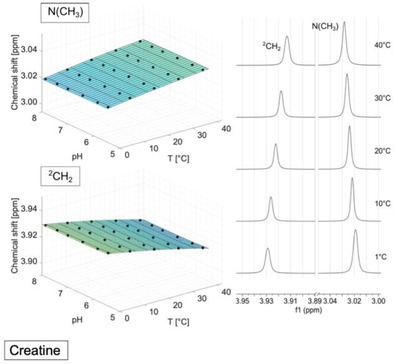

**Abbreviations**
Ala: Alanine
AQSES: Automated quantitation of short echo time MRS spectra
Asp: Aspartate
CEST: Chemical exchange saturation transfer
Cr: Creatine
DSS: 2,2‐Dimethyl‐2‐silapentane‐5‐sulfonate
FASTMAP: Fast automatic shimming technique by map‐ping along projections
GABA: γ‐aminobutyric acid
Gln: Glutamine
Glu: Glutamate
Lac: Lactate
m‐Ins: myo‐inositol
MRS: Magnetic resonance spectroscopy
NA: Number of accumulation
NAA: N‐acetylaspartate
NMR: Nuclear magnetic resonance
PCr: Phosphocreatine
PRESS: Point resolved spectroscopy sequence
QUEST: Quadrupolar Exact Software
RF Radio: frequency
SW: Spectral width
T2: Transversal relaxation time constant
TARQUIN: Totally Automatic Robust Quantitation in NMR
Tau: Taurine
tCr: Total creatine
TE: Echo time
TMS: Tetramethylsilane
TR: Repetition time
TSP: 3‐(trimethylsilyl) propionate
VAPOR: Variable power and optimised relaxation delays

## 1 Introduction

Proton NMR spectroscopy (^1^H MRS) enables the non‐invasive measurement of numerous metabolites in biological systems, both in vitro and in vivo, thereby offering the possibility to study characteristic metabolic processes and identify biomarkers in normal and pathological conditions [1, 2, 3, 4, 5, 6, 7]. In addition, the number of in vivo biomedical applications of ^1^H MRS has steadily increased in recent years, driven by the availability of high magnetic field strengths and improved performance. Therefore, reliable quantification of compounds appearing in the biological system is essential for the relevance of ^1^H MRS. In vivo, ^1^H MRS with a short echo time T_E_ is often preferred for simultaneously detecting a wide range of compounds. However, these sequences have the disadvantage that the analysis of these spectra is frequently hampered due to substantial signal overlaps. For this reason, prior knowledge of chemical shifts and J‐coupling constants is essential for unambiguously identifying the compounds of interest.

Established quantification methods such as LCModel [8], QUEST [9, 10], AQSES [11] or TARQUIN [12] use a model spectrum for each metabolite to minimise the number of variables during the fitting procedure. These model spectra are either measured on phantom solutions or simulated using published values for chemical shifts and J‐coupling constants as prior knowledge. In particular, Govindaraju et al., who analysed the proton NMR chemical shifts and J‐coupling constants for 35 metabolites, have laid an essential basis for generating model spectra for parametric spectral analysis of proton spectra [13, 14].

Quantifying spectra and determining chemical shifts often requires a reference signal, ideally chemically inert and clearly separated from the other signals in the spectrum [15]. Furthermore, the chemical shift of this compound's signal must remain independent of certain variables, such as temperature or pH. For in vitro measurements, the reference compounds tetramethylsilane (TMS), 3‐(trimethylsilyl) propionate (TSP) and 2,2‐dimethyl‐2‐silapentane‐5‐sulfonate (DSS) are widely accepted in organic solvents and aqueous solutions, respectively. DSS is particularly characterised by its temperature and pH‐independent chemical shift [16]. However, since both substances are absent in the in vivo system, other substances must be considered for in vivo applications, for example, the methyl resonances of N‐acetyl aspartate (2.01 ppm) or creatine (3.03 ppm) [15].

In vivo, MRS studies in humans and rodents are usually performed at a body temperature of approximately 37°C. However, pyrexia or anaesthesia significantly affects body temperature, which may rise to 40°C in rodents or drop to 32°C–35°C under anaesthesia, especially without an external system to control body temperature [17]. Furthermore, there is an increasing interest in using alternative animal models for MR studies in experimental medicine or comparative physiology, including birds [18], lower vertebrates such as amphibians [19], fish [20, 21], in particular zebrafish as a model system [22, 23, 24] and invertebrates [25]. The body temperatures of these organisms can vary significantly from those of conventional model organisms, depending on their ambient temperature, ranging from very low temperatures around the freezing point of water [21, 26] to 40°C and higher in insects [27]. Additionally, in vitro ^1^H MRS on extracts is often carried out at temperatures far below those of conventional in vivo applications [28].

Another crucial physiological factor to consider in ^1^H MRS studies is the pH value. The pH plays a crucial role in cellular functions, such as cell growth and proliferation, mitochondrial and enzyme activity and is an essential marker for many pathologies and disease progression [29, 30]. Thus, depending on the physiological conditions, the intracellular pH (pH_i_) value can range from approximately pH 6.5 in the peri‐infarct penumbra to pH 7.7 in tumour cells [29, 30, 31, 32].

^1^H MRS, combined with pattern recognition analysis, can also be used to collect full metabolic profiles and characterise metabolic changes in bacteria [33, 34]. However, depending on the genus, extremophilic bacteria can have pH_i_ values of 4.4–7.8 [35, 36].

Previous studies have investigated the temperature dependent ^1^H chemical shifts of brain metabolism compounds at 7°C and a temperature range of 0°C–40°C [37, 38]. In addition, studies have investigated the influence of temperature on the ^1^H chemical shifts of amide protons [39], proteins [40] and solvents used for reference signals [41, 42, 43]. Furthermore, the pH dependence of some ^1^H MRS signals in the downfield region of the spectra has been demonstrated [13, 14]. Consistent with this, Vermathen et al. presented the pH dependence of the chemical shifts of the histidine resonances [44, 45]. Brey Jr. et al. investigated, among other compounds, the temperature dependence of the coupling constants of vinyl halides and vinyl esters [46]. The proton–proton coupling constant showed no dependence on temperature. Nevertheless, the proton‐fluorine coupling constant decreased with increasing temperature. In another study, the temperature dependence of the ^1^H–^1^H, ^1^H–^19^F and ^19^F–^19^F coupling constants of fluorobenzene and 1,2‐difluorobenzene was measured, resulting in a maximum variation of 5% [47]. However, there is a lack of studies investigating the temperature and pH dependence of coupling constants.

Typical quantification algorithms allow for the correction of frequency shifts, but only as a common correction for all compound resonances. This can lead to quantification problems if the chemical shifts' pH and temperature dependence behave differently. This has already been investigated for the influence of temperature at a constant pH value [38]. However, studies investigating the combined effect of pH and temperature on the chemical shifts and J‐coupling constants of important metabolic compounds are lacking.

Therefore, this study aims to investigate the pH and temperature dependencies of ^1^H chemical shifts and J‐coupling constants not only selectively but also over a broader range of temperatures and pH values. It is important to note that this study focuses on metabolic compounds of particular interest to our in vivo studies, which primarily examine the effects of environmental changes on marine organisms [21].

Therefore, we conducted in vitro measurements on phantoms over a temperature range of 0°C–40°C and a pH range from pH 5.5 to 8.0. Based on the experimentally collected data, the pH and temperature dependence of the chemical shifts and J‐coupling constants.

Subsequently, simulations were conducted to analyse the consequences of spectrum quantification. For this purpose, spectra were simulated for all combinations of pH (7.2, 6.5 and 7.7) and temperature (37°C, 32°C, 35°C and 40°C) to account for both physiologically unremarkable conditions and possible clinical scenarios. Focusing on total creatine (tCr) and the sum of glutamate (Gln) and glutamine (Gln) (Glx) as well as the separate quantification of the contributing metabolites creatine (Cr), phosphocreatine (PCr), Glu and Gln, which play an essential role in the cellular energy status and in several neurological and psychiatric diseases.

## 2 Experimental

### 2.1 Pulse Sequence Parameters

The ^1^H NMR measurements were performed on a wide‐bore 400 MHz NMR spectrometer (9.4 T WB with Avance III HD electronics, Bruker Biospin, Germany) using a triple‐tunable ^1^H, ^13^C, ^15^N NMR probe with a z‐gradient for 1.7 mm NMR tubes. ^1^H spectra were acquired using a standard single‐pulse sequence without water suppression. The following sequence parameters were used: pulse program zg, acquisition time 6.39 s, relaxation delay 15 s, spectral width 8012 Hz, dummy scans 2 and number of scans 16. Sample temperatures were adjusted to 40°C, 30°C, 20°C, 10°C and 1°C using the integrated cooling system of the NMR spectrometer (BCU II, Bruker).

### 2.2 Metabolite Solutions

For the preparation of metabolite solutions, three or four compounds (each with 10 mM concentration) were dissolved in phosphate‐buffered saline (12 mM HPO_4_^2−^), consisting of 90% distilled water and 10% D_2_O (deuterium oxide) containing 0.75 wt. % 3‐(trimethylsilyl)propionic‐2,2,3,3‐d_4_ acid (TSP) as internal standard. Additionally, 2,2‐dimethyl‐2‐silapentane‐5‐sulfonate (DSS) was added as a chemical shift reference (5 mM) [16]. The solutions were titrated to six pH values between 5.5–8.0 (0.1 M NaCl). In each group, only such metabolites were combined that do not cause signal overlap in the spectrum. Solution (1): alanine (Ala), creatine (Cr), taurine (Tau). Solution (2): aspartate (Asp), choline (Cho). Solution (3): γ‐aminobutyric acid (GABA), N‐acetyl aspartate (NAA), glycine (Gly), lactate (Lac). Solution (4): glutamate (Glu), histidine (His), threonine (Thr). Solution (5): glutamine (Gln), myo‐inositol (m‐Ins), phosphocreatine (PCr).

### 2.3 Data Processing and Fitting

Spectra processing and determination of chemical shifts and J‐coupling constants were performed using the software MestReNova 14.2.0 (Mestrelab Research S. L., Santiago de Compostela, Spain). Data processing consisted of zero‐filling to 2048 K complex data points, Fourier transformation and an automatic phase and baseline correction. The chemical shifts were determined relative to the resonance of the trimethyl hydrogen group of DSS [13, 14], which was set at 0.0 ppm regardless of temperature and pH value.

For most metabolites, chemical shifts and J‐coupling constants were determined using automatic peak picking and multiplet analysis based on the global spectral deconvolution (GSD) algorithm [48]. Due to their complex multiplet structure, the metabolites Gln and Glu were processed separately. Data processing was performed using a program in the interactive data language IDL (Research Systems Inc., Boulder, CO, USA). Subsequently, a C++ program using a simplex algorithm determined the chemical shift values. This optimisation procedure minimised the difference between the experimental and fitted spectra calculated by the GAMMA NMR library and used the J‐coupling constants published in [13, 14] as prior knowledge.

### 2.4 Data Modelling

The pH dependence of the chemical shifts and J‐coupling constants was described as a sigmoidal function with the variables a, b and c following a modified form of the Henderson–Hasselbalch equation.

$$\delta ,J\left(\mathrm{pH}\right)=a+\frac{\left(b-a\right)}{1+10^{\left(c-\mathrm{pH}\right)}}$$

Since the temperature (*T* [K]) dependence of chemical shifts can only be assumed to be linear in a small temperature range [49, 50], a 2nd‐degree polynomial dependence was assumed for the investigated range between 0°C and 40°C.

### 2.5 Simulations and Quantifications

The spectra of NAA, Als, GABA, Asp, Cho, Cr, Glu, Gln, Gly, His, m‐Ins, Lac, PCr, Tau and Thr were simulated using the jMRUI software package 6.0beta [51]. As prior knowledge for 37°C and pH 7, the chemical shifts and J‐coupling constants determined in this work were used. Lacking values regarding the coupling constants were completed with the work of Govindaraju et al. and Govind et al. [13, 14].

The chemical shifts and coupling constants were adjusted to the individual temperatures and pH values by exploiting the previously determined models. Assuming strong J‐coupling, spectra were simulated for a symmetric point resolved spectroscopy sequence (PRESS) with TE = 8 ms, 4096 complex data acquisition points and a sampling interval of 0.25 ms [52]. Noise‐free data sets were designed to determine the impact of temperature and pH changes on spectrum quantification.

The metabolites NAA (9 mM), Ala (0.65 mM), GABA (1.5 mM), Asp (2 mM), Cho (1 mM), Cr (4 mM), Gln (3 mM), Glu (8 mM), Gly (0.6 mM), His (0.1 mM), Lac (1.3 mM), m‐Ins (6.2 mM), PCr (4.5 mM), Tau (6 mM) and Thr (0.3 mM) were simulated with a typical in vivo line width of 8 Hz [3]. The metabolite concentrations were adjusted to mimic a rat brain [4, 5, 53].

In order to analyse the potential influence of, e.g., pyrexia and experimentally induced cooling, datasets were simulated for temperatures of 40°C, 37°C, 35°C and 32°C. In addition, these datasets were modelled for pH values of 6.5, 7.2 and 7.7 to investigate the effects of, e.g., peri‐infarct and tumour cells. The temperature‐ and pH‐dependent spectra were analysed using the time‐domain quantification method AQSES [11], as provided by jMRUI 6.0beta. The basis sets of metabolite profiles were simulated for the upfield range.

## 3 Results

### 3.1 Temperature and pH‐Dependent Chemical Shifts of Brain Metabolites

The spectra of all metabolites are briefly presented at the beginning of each section, facilitating an understanding of the results. Detailed explanations, including structural formulae, characterisation of the spin system and spectrum and a more detailed description of the metabolite, can be found in the works of Govindaraju et al. [13], Govind et al. [14] and de Graaf [15]. In the following, we refer to the nomenclatures introduced in these papers. For a clear presentation of the spectra, the line‐fitted resonances for the respective metabolites are shown.

The results section displays only two signals showing the most significant temperature and pH‐dependent changes for each compound. An overview of all resonances of a compound, including example spectra and J‐coupling constants, is given in the supplementary material (Figures S1–S15). The parameters of the calculated model functions $\delta \left(\mathrm{pH},T\right)$ and $J\left(\mathrm{pH},T\right)$ can also be found there (Tables S1 and S2). The chemical shifts are given relative to DSS at 0 ppm. Model functions for rapidly exchanging proton groups of some metabolites could not be generated because these signals were only detectable at very low temperatures and acidic pH and thus too few data points were available for a reasonable fit. In addition, we have summarised the direction and magnitude of the changes in chemical shift and J‐coupling constants as a function of temperature and pH in the tables (Tables S1, S2.

From the repeated alanine measurements, we estimated a standard deviation for the determined chemical shifts of the doublet and quartet ±0.0001 ppm and for the J‐coupling constants of ±0.003 Hz.

### 3.2 N‐Acetylaspartate (NAA)

**FIGURE 1:**
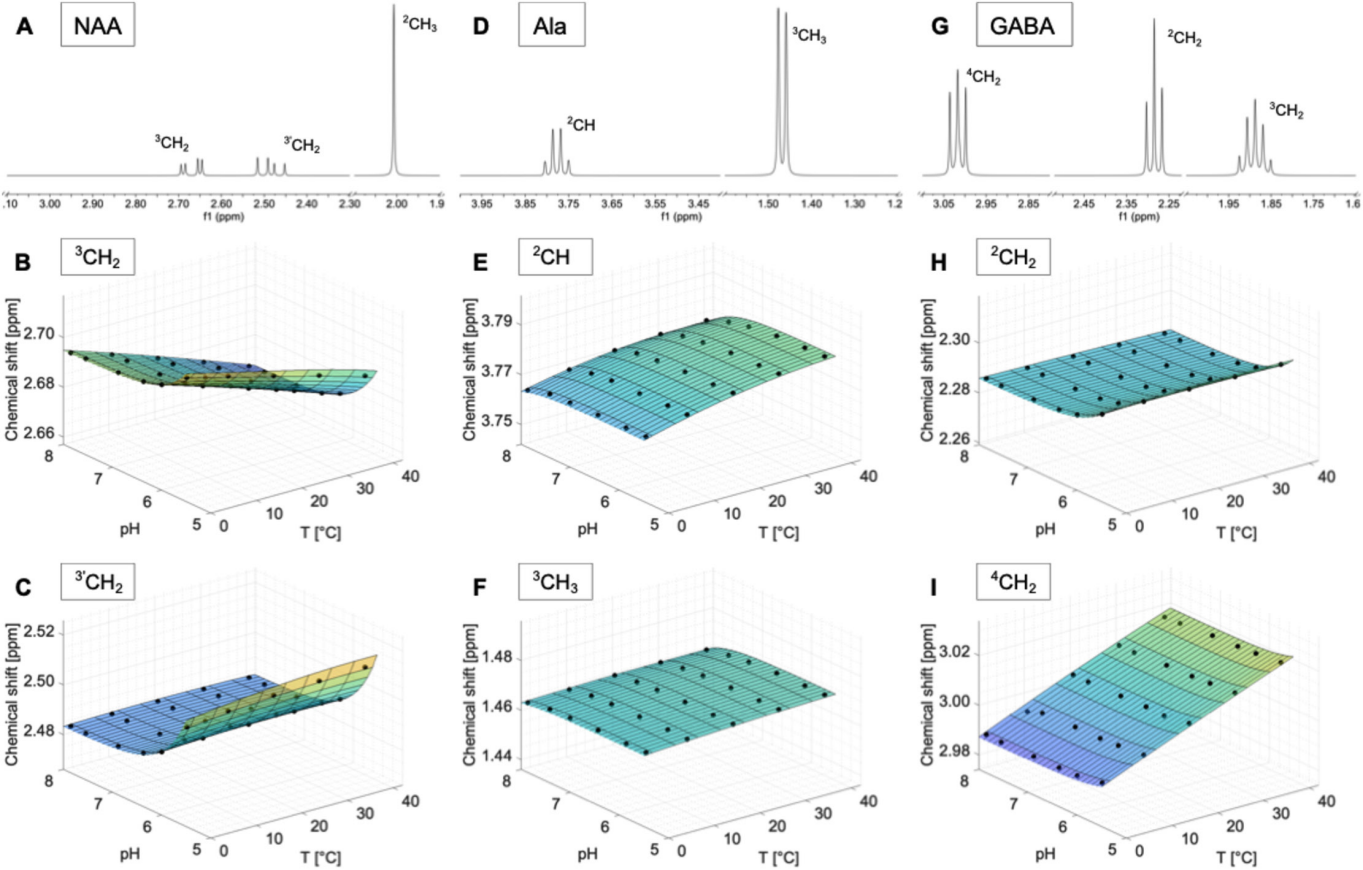
Spectra measured on a 400 MHz spectrometer referenced to DSS (pH 7.0, 40°C) of N‐acetylaspartate (NAA) (A), alanine (Ala) (D) and *γ*‐Aminobutyric acid (GABA) (G). Additionally, the chemical shifts as a function of pH and temperature and the calculated model functions $\delta \left(\mathrm{pH},T\right)$ for the two resonances of each compound that show the strongest dependence on temperature and/or pH are displayed. Subfigures B, C illustrate the ^3^CH_2_ and ^3'^CH_2_ resonance of NAA. E, F show the dependencies of the chemical shifts on pH and temperature for the ^2^CH and ^3^CH_3_ resonance of Ala. The subfigures H, I illustrate the ^2^CH_2_ and ^4^CH_2_ resonances of GABA.

The most prominent resonance in the NAA spectrum is the methyl group at 2.01 ppm (Figures 1A and S1A). Smaller resonances appear as doublet‐of‐doublets at 2.49, 2.67 and 4.38 ppm, corresponding to the protons of the aspartate CH_2_ and CH groups. The NH proton of the amide produces a broad doublet at 7.82 ppm.

The chemical shift of the singlet of the ^2^CH_3_ group is almost constant in the whole temperature and pH range (Figure S1B,D). The resonances of the ^3'^CH_2_ group also show nearly no temperature dependence but a pH dependence, which is expressed in a shift of the resonances in the direction of the water signal for acidic pH values (Figures 1C and S1G). Additionally, the ^3^CH_2_ group exhibits an almost linear drift in chemical shift with temperature (Figures 1B and S1F). The ^2^CH group could only be evaluated up to a temperature of 30°C, due to its close proximity to the water signal. This group exhibits only minor dependence of the chemical shift on temperature, but a strong drift of the chemical shift towards downfield with increasing acidity, particularly at high temperatures (Figure S1E). No significant changes were observed in the line shapes as a function of temperature and pH. However, moderate changes in the J‐coupling constants were found (Figure S1B,I–K).

The signal of the NH group shows a linear shift of the resonance as a function of temperature, independent of pH (Figure S1H). Furthermore, a change of the line shape as a function of temperature from a clearly formed doublet to a broad singlet with increasing temperature can be observed (Figure S1C).

### 3.3 Alanine (Ala)

The spectrum of alanine shows resonances of two proton groups (Figures 1D and S2A). The ^3^CH_3_ group forms a doublet at 1.47 ppm and the ^2^CH group shows a quartet at 3.77 ppm.

Both proton groups exhibit no change in chemical shift with varying pH (Figures 1E,F and S2B,D,E). The ^2^CH group shows a moderate shift of the quartet towards downfield with increasing temperature (Figures 1E and S2C,D). No changes in the line shapes or the J‐coupling constant of the resonances were found as a function of temperature or pH (Figure S2B,C,F).

### 3.4 γ‐Aminobutyric Acid (GABA)

The spectrum of GABA is composed of three different multiplets corresponding to the three methylene groups at 1.89 (^3^CH_2_), 2.28 (^2^CH_2_) and 3.01 ppm (^4^CH_2_) (Figures 1G and S3A) [54].

The ^3^CH_2_ group shows no change in chemical shift as a function of temperature and pH (Figure S3D,B). In comparison, the ^2^CH_2_ group exhibits a slight dependence of the chemical shift on the pH value, which is manifested by a slight drift towards downfield in the acidic range (Figures 1H and S3C). In contrast, the chemical shift of the ^4^CH_2_ group exhibits a strong temperature dependence, resulting in a drift of the chemical shift towards the downfield direction with increasing temperature (Figures 1I and S3E). In addition, the shape of the individual signals of the multiplet becomes sharper with increasing temperature (Figure S3B,F).

### 3.5 Aspartate (Asp)

**FIGURE 2:**
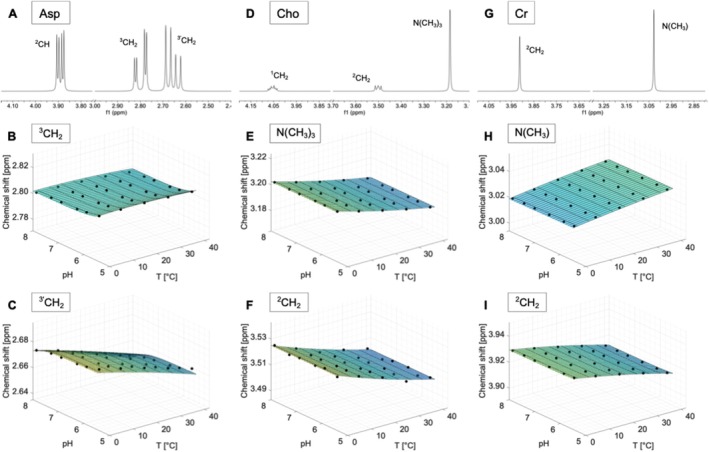
Spectra measured on a 400 MHz spectrometer referenced to DSS (pH 7.0, 40°C) of aspartate (Asp) (A), choline (Cho) (D) and creatine (Cr) (G). Additionally, the chemical shifts as a function of pH and temperature and the calculated model functions $\delta \left(\mathrm{pH},T\right)$ for the two resonances of each compound that show the strongest dependence on temperature and/or pH are displayed. Subfigures B, C show the dependencies of the chemical shifts on pH and temperature for the ^3^CH_2_ and ^3'^CH_2_ resonance of Asp. In subfigure E, F the N (CH_3_)_3_ and ^2^CH_2_ resonances of choline and their model functions are shown. H, I illustrate the N (CH_3_) and ^2^CH_2_ resonance of Cr.

The spectrum of aspartate shows a doublet‐of‐doublets from the CH group at 3.89 ppm and a pair of doublet‐of‐doublets from the protons of the ^3^CH_2_ group at 2.65 and 2.80 ppm (Figures 2A and S4A).

The ^3'^CH_2_ group shows a change in chemical shift as a function of temperature, such that the resonance shifts upfield with increasing temperature (Figures 2C and S4F). In addition, the J‐coupling constants also show a temperature dependence (Figure S4G–I). The proton groups ^2^CH and ^3^CH_2_ show no change in chemical shift as a function of pH and temperature (Figures 2B and S4B–E).

### 3.6 Choline (Cho)

The spectrum of choline consists of a prominent singlet at 3.2 ppm from the N (CH_3_)_3_ group (Figures 2D and S5A). Furthermore, the ^1^CH_2_ and ^2^CH_2_ groups form multiplets centred at 4.05 and 3.50 ppm, respectively.

The chemical shifts of the N (CH_3_)_3_ and ^2^CH_2_ groups exhibit a very similar dependence on temperature, resulting in an upfield drift with increasing temperature (Figures 2E,F and S5B,C,E). However, no changes in the line shape (Figure S5B) and no dependence on pH can be observed (Figure S5C,E). The chemical shift of the ^1^CH_2_ group is almost independent of temperature and pH (Figure S5D).

### 3.7 Creatine (Cr)

The spectrum of creatine consists of a very prominent singlet of the methyl protons at 3.03 ppm and the methylene protons form a singlet at 3.91 ppm (Figures 2G and S6A). The resonance of the NH protons shows a peak at 6.65 ppm.

All proton groups of creatine exhibit a temperature‐dependent shift in their chemical shifts, with the groups shifting in opposite directions. While the N (CH_3_) group shifts towards downfield with increasing temperature (Figures 2H and S6B,D), the ^2^CH_2_ and NH groups shift towards upfield (Figures 2I and S6E,F). In addition, a change in the line shape of the NH resonance as a function of pH from a narrow to a broad singlet with a more basic pH can be observed (Figure S6C).

### 3.8 Glutamate (Glu)

**FIGURE 3:**
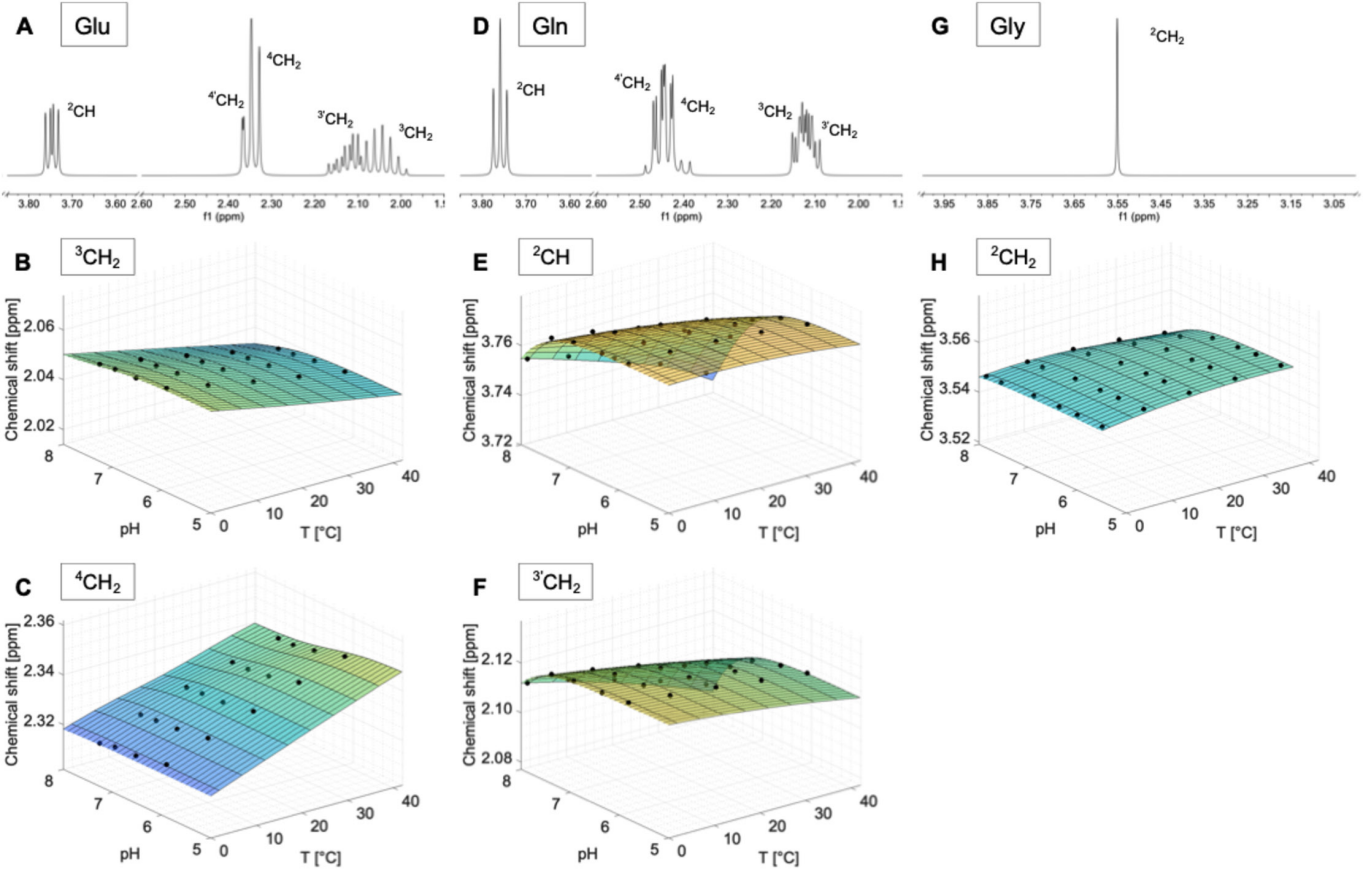
Spectra measured on a 400 MHz spectrometer referenced to DSS (pH 7.0, 40°C) of glutamate (Glu) (A), glutamine (Gln) (D) and glycine (Gly) (G). Additionally, the chemical shifts as a function of pH and temperature and the calculated model functions $\delta \left(\mathrm{pH},T\right)$ for the two resonances of each compound that show the strongest dependence on temperature and/or pH are displayed. Subfigures B, C, E and F show the dependencies of the chemical shifts on pH and temperature for the ^3^CH_2_ and ^4^CH_2_ resonance of Glu and the ^2^CH and ^3'^CH_2_ signals of Gln, respectively. The subfigure H illustrates the pH and temperature dependence of the ^2^CH_2_ singlet of Gly and the corresponding model function $\delta \left(\mathrm{pH},T\right)$.

The complex spectrum of glutamate consists of a doublet‐of‐doublets centred at 3.74 ppm, while the resonances from the two methylene groups are grouped in the 2.04–2.35 ppm range (Figures 3A and S7A).

All chemical shifts of the resonances in the spectrum of glutamate show a dependence on temperature and only slight changes as a function of pH (Figures 3B,C and S7B–G). However, the signals drift in opposite directions as the temperature increases. While the ^2^CH and ^3^CH_2_ signals shift upfield with increasing temperature (Figures 3B and S7C,D), the ^3'^CH_2_, ^4^CH_2_ and ^4'^CH_2_ signals shift downfield (Figures 3C and S7E–G). Furthermore, concise changes in the line shape of the signals can be observed with temperature changes, which are reflected in a sharpening of the signal with increasing temperature (Figure S7B).

### 3.9 Glutamine (Gln)

The spectrum of glutamine is structured very similarly to that of glutamate. A triplet from the methine proton resonates at 3.75 ppm (Figures 3D and S8A) and the multiplets from the four methylene protons are grouped from 2.12 to 2.46 ppm. The two amide protons appear at 6.82 and 7.53 ppm.

The resonances of the ^2^CH and ^3'^CH_2_ proton groups mainly show a dependence of the chemical shift on pH (Figures 3E,F and S8B,D,F). However, the chemical shifts of the ^3^CH_2_, ^4^CH_2_ and ^4'^CH_2_ proton groups shift towards the downfield region with increasing temperature (Figure S8E,G,H). The two NH_2_ groups exhibit a similar temperature dependence and their resonances shift upfield with increasing temperature (Figure S8C,I,J). Furthermore, a temperature dependence of the line shape of the NH_2_ groups is observed, i.e., a sharpening of the signal with decreasing temperature (Figure S8C).

### 3.10 Glycine (Gly)

The simple spectrum of glycine consists of a single singlet of the two methylene protons at 3.55 ppm (Figures 3G and S9A).

The signal shows only slight dependence of the chemical shift on pH and temperature (Figures 3H and S9B,C). However, a change in line shape can be observed as a function of temperature, which manifests in a broadening of the signal with decreasing temperature (Figure S9B).

### 3.11 Histidine (His)

**FIGURE 4:**
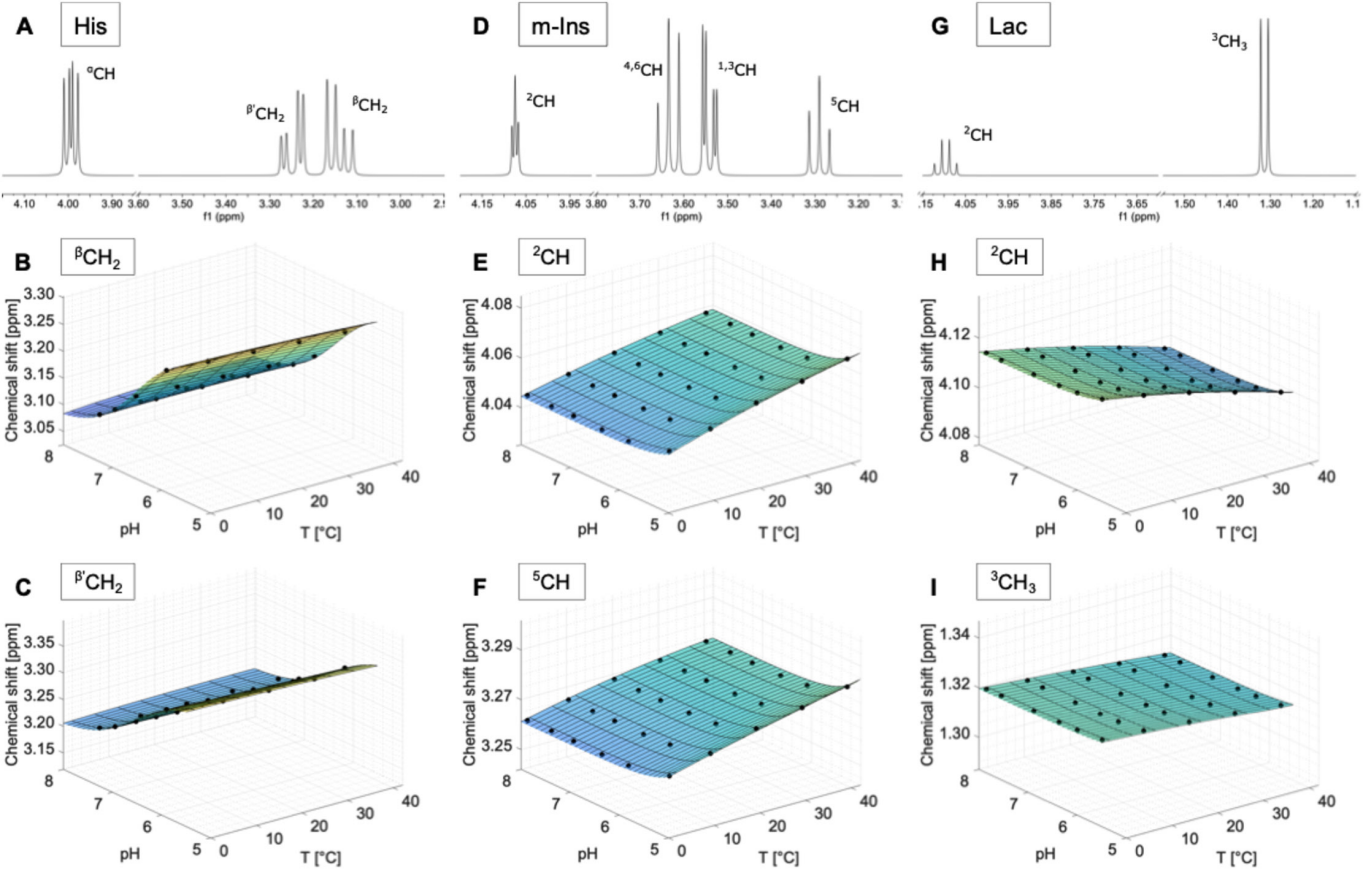
Spectra measured on a 400 MHz spectrometer referenced to DSS (pH 7.0, 40°C) of histidine (His) (A), myo‐Inositol (m‐Ins) (D) and lactate (Lac) (G). Additionally, the chemical shifts as a function of pH and temperature and the calculated model functions $\delta \left(\mathrm{pH},T\right)$ for the two resonances of each compound that show the strongest dependence on temperature and/or pH are displayed. Subfigures B, C show the dependencies of the chemical shifts on pH and temperature for the ^β^CH_2_ and ^β'^CH_2_ resonance of His. In subfigures E, F the ^2^CH and ^5^CH resonances of m‐Ins and their model functions are shown. H, I illustrate the ^2^CH and ^3^CH_3_ resonance of Lac.

The spectrum of histidine consists of three doublet‐of‐doublets centred at 3.98 ppm (^α^CH), 3.12 (^β^CH_2_) and 3.22 (^β'^CH_2_) in the upfield part of the spectrum (Figures 4A and S10A). The water‐exchangeable imidazole protons show resonances at 7.8 and 7.1 ppm (Figure S10C).

All resonances seen in the spectrum of histidine show a different dependence on the pH value, which is reflected in a drift of the signals towards the upfield region, while the temperature dependence is negligible (note the different scaling compared to the other metabolites) (Figures 4B,C and S10B–H). In addition, a change in line shape of the doublet‐of‐doublets can be observed and also in the J‐coupling constants (Figure S10B,I–K). It should be noted that the two resonances ^β^CH_2_ and ^β'^CH_2_, with a more acidic pH value, combine to form a multiplet (Figure S10B).

### 3.12 Myo‐Inositol (m‐Ins)

The spectrum of myo‐inositol is composed of four distinct groups of resonances (Figures 4D and S11A). The ^1,3^CH group forms a doublet‐of‐doublets, which is centred at 3.52 ppm. The resonances of the ^5^CH, ^4,6^CH and ^2^CH groups appear as triplets in the spectrum at 3.27, 3.61 and 4.05 ppm, respectively.

The ^1,3^CH group exhibits a slight shift in the signal towards the water signal with increasing acidity, while the chemical shift as a function of temperature remains constant. The ^2^CH, ^4,6^CH and ^5^CH proton groups of myo‐inositol show an almost identical behaviour, with a moderate drift of the chemical shift towards the water signal with increasing temperature and a similar change of the chemical shift as a function of pH as the ^1,3^CH group (Figures 4E,F and S11C–F). The resonances exhibit no changes in line shape with varying pH (Figure S11B).

### 3.13 Lactate (Lac)

The spectrum of lactate consists of a methyl group that shows a doublet at 1.31 ppm and a methine group that exhibits a quartet centred at 4.09 ppm (Figures 4G and S12A).

Both groups exhibit a slight dependence of the chemical shift on temperature, which is evident in a drift away from the water signal with increasing temperature (Figures 4H,I and S12C,D). In addition, small changes in the line shape can be observed, specifically a narrowing of the signal with increasing temperature (Figure S12B) and a slight change in the J‐coupling constants (Figure S12E).

### 3.14 Phosphocreatine (PCr)

**FIGURE 5:**
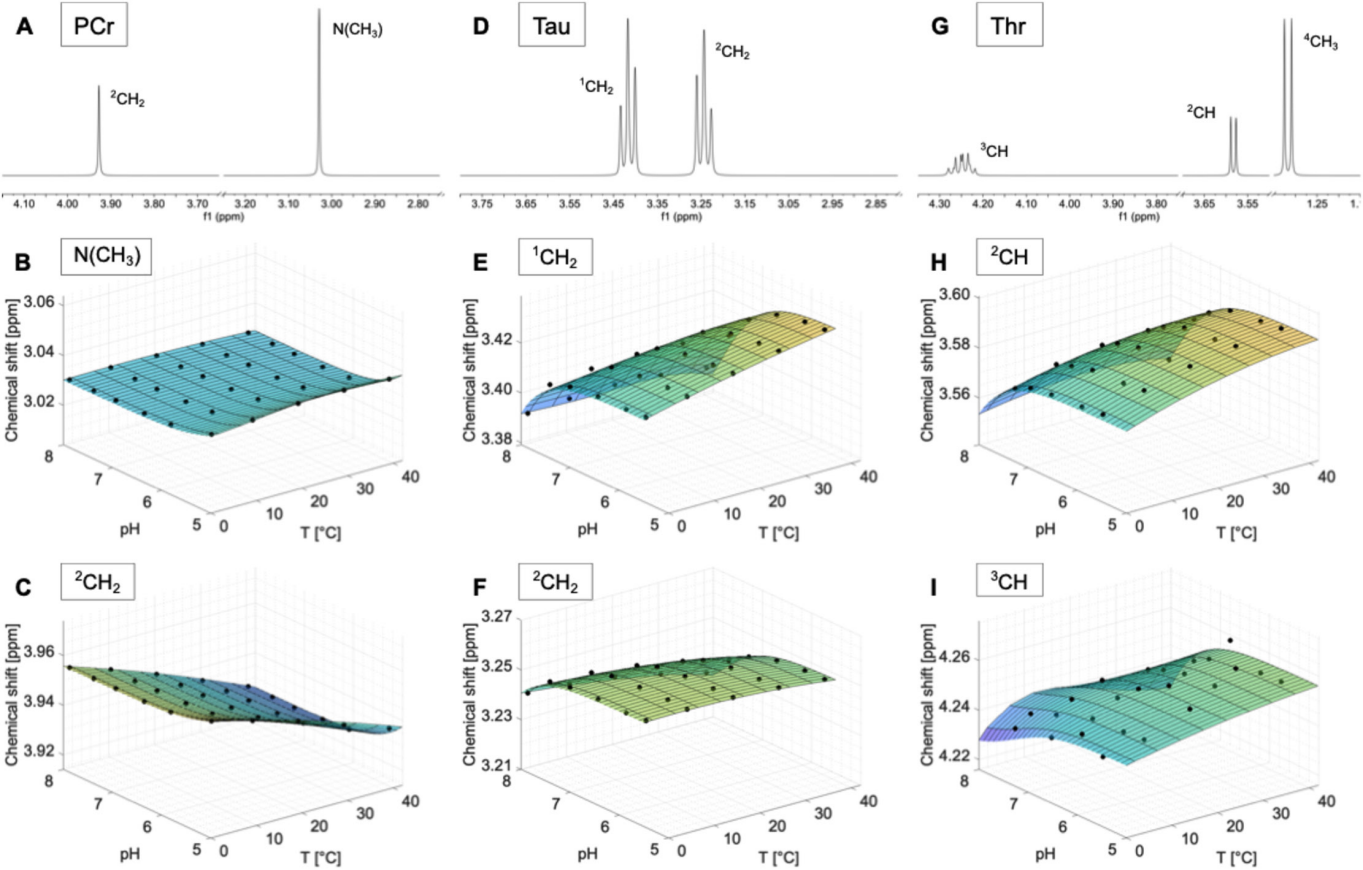
Spectra measured on a 400 MHz spectrometer referenced to DSS (pH 7.0, 40°C) of phosphocreatine (PCr) (A), taurine (Tau) (D) and threonine (Thr) (G). Additionally, the chemical shifts as a function of pH and temperature and the calculated model functions $\delta \left(\mathrm{pH},T\right)$ for the two resonances of each compound that show the strongest dependence on temperature and/or pH are displayed. Subfigures B, C show the dependencies of the chemical shifts on pH and temperature for the N (CH_3_) and ^2^CH_2_ resonance of PCr. The subfigures E, F illustrate the ^1^CH_2_ and ^2^CH_2_ resonance of Tau. In H and I the chemical shifts and the corresponding model functions of the ^2^CH and the ^3^CH resonance of threonine are displayed.

The spectrum of phosphocreatine shows a similar structure to that of Cr and has a prominent singlet of methyl‐protons at 3.0 ppm and one resulting from the methylene‐protons at 3.9 ppm (Figures 5A and S13A). In addition, the spectrum shows two resonances of the NH protons in the downfield region at 6.6 and 7.3 ppm (Figure S13A).

The chemical shift difference between the two upfield signals decreases with increasing temperature (Figures 5B,C and S13B,D,E). Additionally, a slight decrease in the chemical shift is observed for both resonances in the acidic pH range. The two resonances of the NH protons show a drift towards the water signal with increasing temperature (Figure S13C,F,G). In addition, a substantial change in the line shape can be observed for the resonances of the NH protons. The resonance at approximately 6.6 ppm exhibits a significantly sharper and higher signal in the low‐temperature range compared to 40°C. However, the line shape of the NH resonance around 7.3 ppm also changes in width and intensity with temperature (Figure S13C).

### 3.15 Taurine (Tau)

The spectrum of taurine shows two triplets at 3.25 and 3.42 ppm (Figures 5D and S14A).

The chemical shifts of both triplets exhibit a slight dependence on temperature and a stronger dependence on pH, as evidenced by the uneven shift of the two signals towards the water signal at acidic pH values (Figures 5E,F and S14 B,C,E). No changes in the line shape can be detected.

### 3.16 Threonine (Thr)

The threonine spectrum comprises signals from a CH_3_ and two CH groups (Figures 5G and S15A). The ^4^CH_3_ protons give a doublet at 1.32 ppm. The ^2^CH proton gives a doublet at 3.58 ppm and the ^3^CH proton resonates at 4.25 ppm.

The chemical shift of the doublet of the ^2^CH group shows a slight dependence on temperature and a stronger one on pH (Figures 5H and S15B,C). However, this dependence decreases for the chemical shifts of the ^3^CH and ^4^CH_3_ groups (Figures 5I and S15B,D,E). For the J‐coupling constants, no significant changes can be observed with varying pH or temperature (Figure S15F,G).

### 3.17 Quantifying Brain Metabolites From Simulated Data Sets for Different Temperatures and pH Values

**FIGURE 6:**
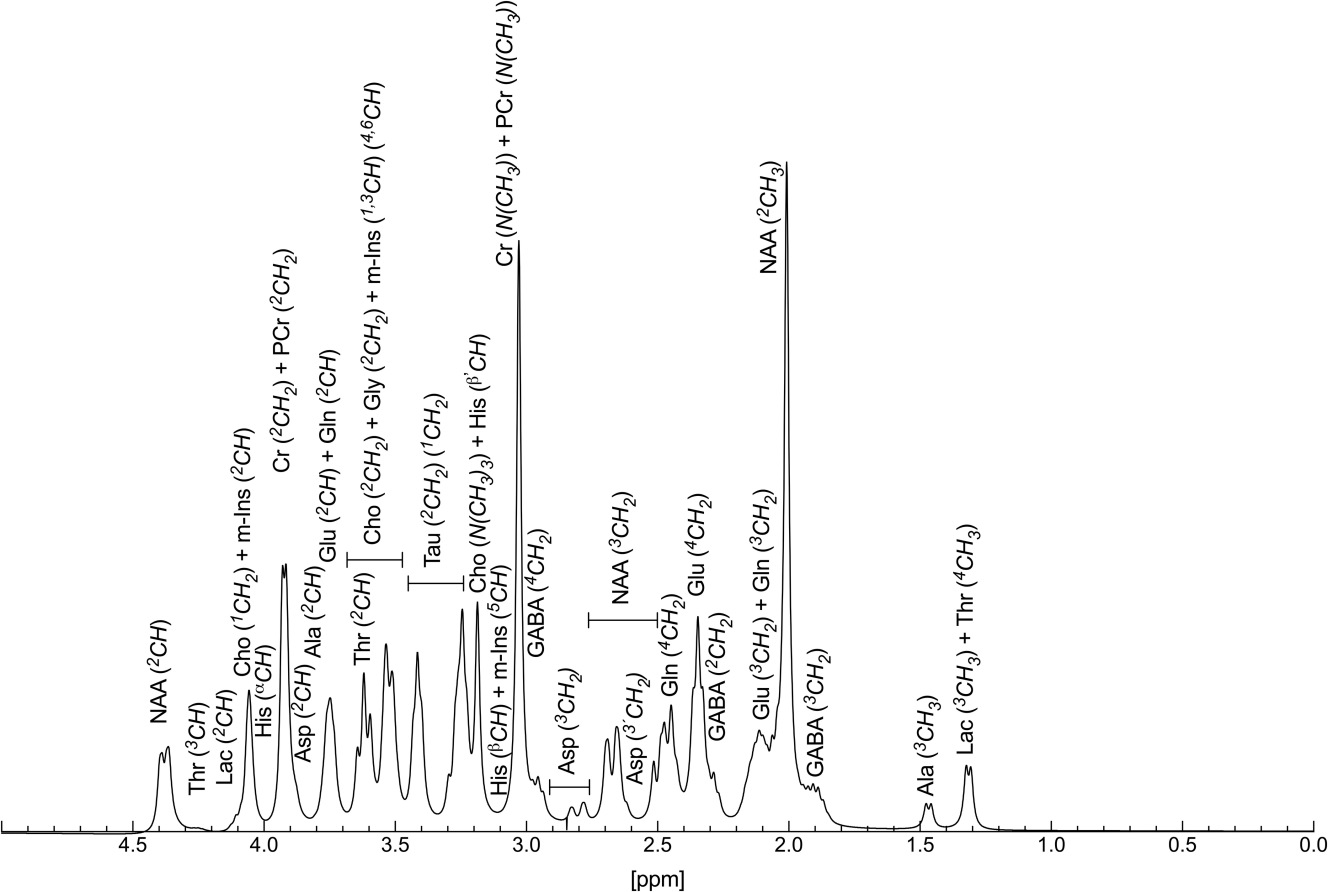
^1^H NMR spectrum with the examined metabolites simulated for 37°C and a line width of 8 Hz (pH 7.2).

Figure 6 illustrates a typical ^1^H NMR spectrum with the examined metabolites simulated for 37°C and pH 7.2. The signals are assigned to the metabolites and the corresponding proton groups.

The percentage concentrations of the analysed metabolites determined using the AQSES algorithm can be found in the supplement (Figure S16A–O). The spectra, composed of the analysed metabolites, were simulated at different temperatures (32°C–40°C) and pH values (6.5–7.7), using typical metabolite concentrations. Examples of simulated spectra and the resulting residual spectra for temperature variation at constant pH and pH variation at a fixed temperature are provided in the supplement (Figures S17 A–D and S18A–C).

For the data set simulated for 37°C and pH 7.2, most metabolite concentrations show a maximum deviation of 1.5% using the AQSES algorithm, with chemical shift values for 37°C and pH 7 as prior knowledge. Gly is an exception, whose concentration is underestimated by 5.5% (Figure S16I).

For the pure influence of the pH value on the estimated concentration, the results for pH 6.5 (37°C) show an average deviation of 2%. The most significant deviations are shown for Gly (Figure S16I), which is overestimated by 8.5% and His (Figure S16J), which is underestimated by about 11%. An increase in the pH value to 7.7 (37°C) results in an average deviation of 8% from the simulated concentration, excluding that of His. The most considerable deviations, besides His, are shown by Thr and Cho, which are underestimated by 37.5% and 18%, respectively (Figure S16O,E).

**FIGURE 7:**
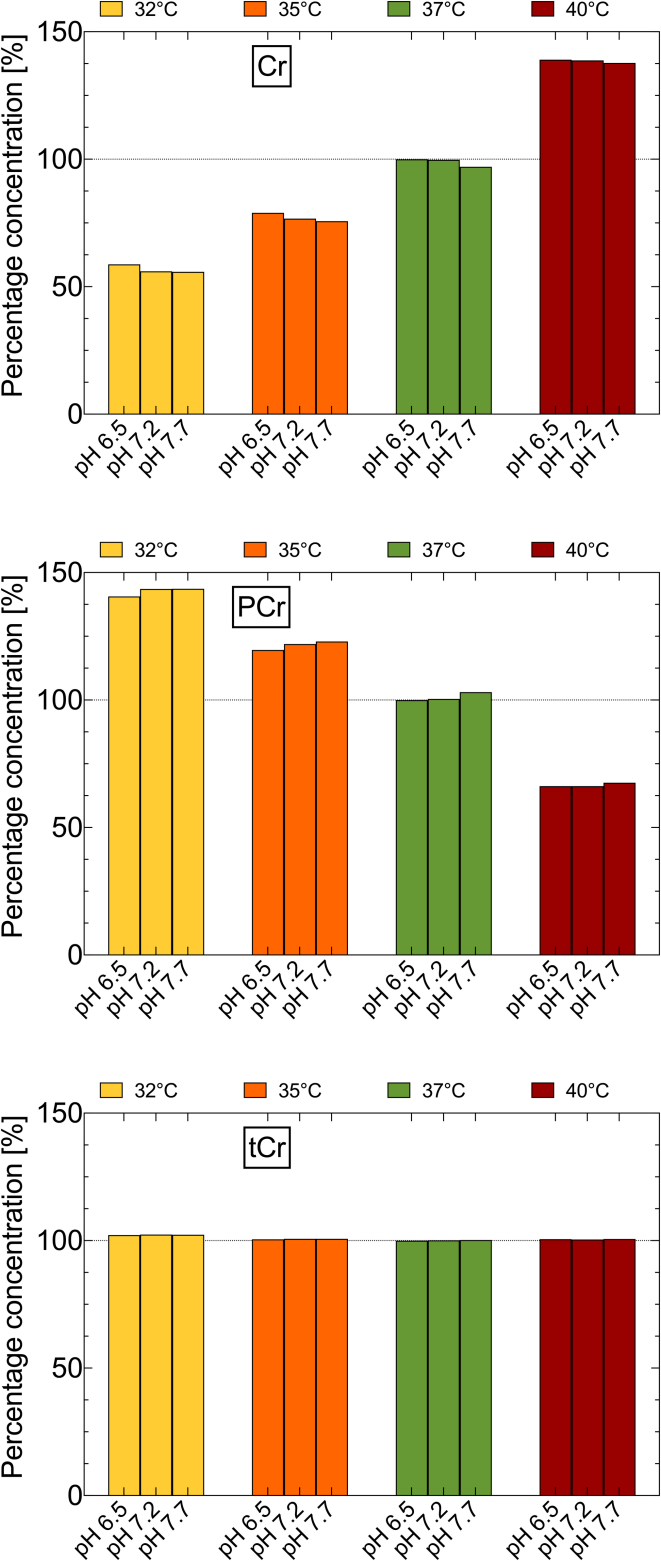
Metabolite concentrations determined by the AQSES algorithm for Cr (A), PCr (B) and the sum signal tCr (C). The results are given in percent of the simulated values.

Lowering the temperature to 32°C (pH 7.2) results in an average deviation of 15% in the estimated percentage concentration (His not included). The most significant deviations are evident in Cr and PCr, which are underestimated by 44% and overestimated by 44%, respectively (Figures 7A,B and S16F,M). Gly and Thr also show considerable deviations of 35% and 25% (Figure S16I,O). For an increase in temperature to 40°C, all metabolites analysed show an average deviation of 11%.

Cr and PCr exhibit opposite trends at higher temperatures compared to lower temperatures. Cr is overestimated by 39% and PCr is underestimated by 34% (Figures 7A,B and S16F,M). In addition, both Gly and His are underestimated by 28% each (Figure S16I,J).

### 3.18 Quantifying tCr and Glx From Simulated Data Sets for Different Temperatures and pH Values

**FIGURE 8:**
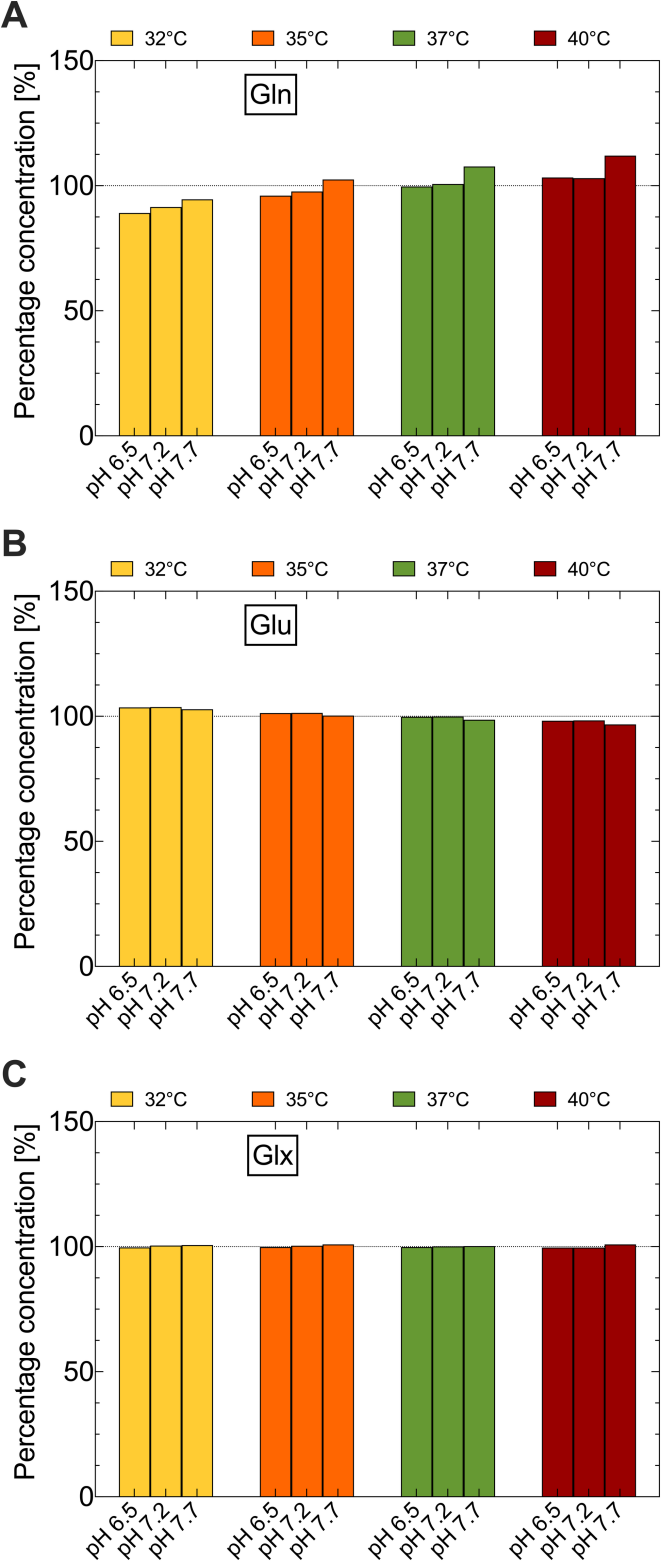
Metabolite concentrations determined by the AQSES algorithm for Gln (A), Glu (B) and the sum signal Glx (C). The results are given in percent of the simulated values.

Figures 7 and 8 present the quantification results for Cr, PCr and tCr as well as Glu, Gln and Glx, using the AQSES algorithm, with chemical shift values for 37°C and pH 7.0 as prior knowledge. The percentage values concerning the simulated concentrations are shown for different temperatures and pH values.

For all pH values in the high‐temperature range, the Cr concentration was overestimated by up to 39% at 40°C and underestimated by up to 44% for temperatures lower than 37°C (Figure 7A). In contrast, PCr showed the opposite tendency (Figure 7B). However, the tCr signals showed only minor deviations for all temperatures and pH values, with a maximum overestimation of about 2% at the lowest temperatures and pH 7.7 (Figure 7C).

For all temperatures and pH values of 7.2 and 6.5, the concentrations of Gln and Glu deviated by a maximum of 4% (Figure 8A,B). However, the deviation of the Glx signal is negligible for all temperatures and these two pH values (Figure 8C). At a pH value of 7.7, the Gln concentration was overestimated by up to 12% for temperatures higher than 35°C and underestimated by about 5.5% for 32°C (Figure 8A). A contrary tendency is evident in the concentration of Glu (Figure 8B). However, even for a pH value of 7.7, the deviation of the Glx signal is negligible (Figure 8C).

## 4 Discussion

The study aimed to investigate the temperature and pH dependence of ^1^H chemical shifts and J‐coupling constants so that their use in model spectra generation can support the correct assignment of resonances and determination of concentrations over a wide temperature and pH range. Therefore, essential metabolic compounds were investigated within a pH range of 5.5–8.0 and at temperatures ranging from 1°C to 40°C. From the 15 metabolic compounds analysed, 10 compounds show significant dependencies on temperature and only three compounds show remarkable changes regarding their dependence on the chemical shift on pH. Gln and NAA depend significantly on temperature and pH, although they exhibit different affinities for the various proton groups.

DSS dissolved in D_2_O at 25°C shows no dependence on the pH value (pH 2–11) [16]. Therefore, this study referenced all chemical shifts to DSS as a standard. This is in contrast to the standard TSP (3‐(trimethylsilyl)‐propionate), which shows a pH dependency and is also widely used. Furthermore, since the temperature dependencies of the chemical shifts of DSS and TSP in D_2_O are reported to be very small, both reference signals are almost immune to temperature variations [16].

The standard deviations estimated from repeated measurements for the determined chemical shifts and J‐coupling constants are comparable to the values reported by Govindaraju et al. However, in the present study, the evaluation is based only on peak picking and a multiplet analysis and no fitting of the data with model functions was performed [13, 14]. This is also the reason why only those chemical shifts and J‐coupling constants that could be determined based on this simplification are given in the present study. However, this simplification reflects the in vivo situation and will, therefore, not affect the assignment of resonances or the determination of concentrations.

Comparing the results of the present study with the literature shows that, for example, the resonance of the NH group of NAA exhibits the characteristic linear chemical shift as a function of temperature, independent of the pH value. Arús et al. determined a slope of 0.00789 ppm/°C [39] in their study. The present study obtained a slope of 0.00881 ppm/°C. The difference can partially be attributed to the different referencing of the signal, which in the work of Arús et al. was based on the N (CH_3_) resonance of Cr, whose chemical shift is also temperature‐dependent. Furthermore, slight differences in the absolute chemical shifts between the values measured in this study and those in other studies or between values measured in solutions and those observed in the cytosol or in vivo, have also been noted in other studies [13, 14, 39]. Other possible reasons for differences in absolute values may be the ionic strength and the concentration of proteins, free magnesium and calcium in the medium [55, 56]. In addition, Govindaraju et al. have shown that the absolute chemical shifts of compounds change, albeit slightly, depending on the solvent [13, 14]. Therefore, it is more important to focus on the differences in the chemical shifts between the different resonances of a metabolite. This is also supported by the fact that the differences in chemical shifts are crucial for quantifying spectra using the established quantification methods mentioned above. Moreover, such deviations between values measured in solutions, cytosol or in vivo could not be observed for J‐coupling constants [13, 14].

In addition to the changes in chemical shifts with changes in temperature and pH known from the literature, e.g., for the NH resonance of NAA [29] and the ^2^CH and ^5^CH signals of histidine [34, 35], the strong pH dependence of the chemical shift of the Tau triplets is particularly notable. The potential use of this dependence to determine intracellular pH values has already been investigated, yielding promising results [42]. Fundamental changes in the covalent structure of the metabolites examined, depending on temperature and pH, are not to be expected, as shown in studies by Silverstein et al. [57] and Bhinderwala et al. [58].

The impact that incorrect prior knowledge can have on the quantification of spectra has already been shown for the temperature dependence of chemical shifts [38]. In a previous study, only slight deviations in the quantification of important brain metabolites were observed for a temperature range of 32°C–40°C. However, determining the absolute concentration of Cr and PCr proved highly susceptible to temperature changes. Furthermore, incorrect quantification of spectra at lower temperatures of 10°C and 1°C could be observed for almost all metabolites without corrected prior knowledge.

This influence of temperature on the absolute quantification of concentration is also reflected in the data collected in this study, while the impact of varying pH values can generally be classified as less important. The influence on quantification due to a chemical shift based on changes in pH value is particularly evident in the metabolites Cho, Gly, Thr and His. Since no significant changes in the chemical shifts of the signals of these metabolites could be observed as a function of pH, it can be assumed that the erroneous quantification can be attributed to the low concentration or overlap with signals from other metabolites, such as m‐Ins [28]. It is important to note that metabolites that showed significant changes in the chemical shift of their signals as a function of pH, such as NAA and Gln, showed no or only minor errors in absolute quantification.

For in vivo quantification of metabolites, an internal standard is necessary, which should be independent of external variables such as temperature and pH [15]. Commonly used internal references are the methyl resonance of NAA (2.01 ppm) and the resonance of the methyl protons of Cr (N (CH)_3_) at 3.03 ppm. The creatine peak is relatively stable with no changes reported with age or a variety of diseases. However, decreased levels are observed in tumours and stroke and increased levels are monitored with myotonic dystrophy [12]. A script written in Scilab (Version 6.0.1, Scilab Enterprises, Versailles, France) that enables referencing the chemical shifts of metabolites to the methylene resonances of NAA and Cr can be provided upon request.

In order to avoid systematic quantification errors when using simulated model functions, it should be noted that, in addition to the temperature dependence of chemical shift and J‐coupling constants, the spin–lattice and spin–spin relaxation times (T_1_ and T_2_) are also temperature‐dependent [15, 59, 60, 61], with T_1_ usually increasing with rising temperature, while the apparent T_2_ values decrease due to the greater influence of diffusion effects. However, the preferred use of pulse sequences with long repetition times and short echo times minimises the influence of T_1_ and T_2_ on quantification and, consequently, their temperature dependence.

The present study emphasises the necessity to ensure that correct prior knowledge is used for quantifying in vivo ^1^H MRS data, which is particularly important when physiological factors such as pH and temperature change, e.g., under pathophysiological conditions.

## 5 Conclusion

The present work provides a detailed compilation of ^1^H chemical shifts and J‐coupling constants of 15 important metabolic compounds, along with the modelling of their pH and temperature dependence. The results can significantly supplement the prior knowledge required to create correct model functions for quantifying in vivo ^1^H MRS data. Thus, our work can contribute to avoiding or minimising quantification errors, which incorrect prior knowledge would cause, especially in cases of variations or changes in pH or temperature, whether due to physiological conditions, the model organism or experimental conditions.

## Author Contributions

**Felizitas C. Wermter:** conceptualisation, methodology, software, formal analysis, investigation, writing – original draft, visualisation, funding acquisition. **Wolfgang Dreher:** conceptualisation, writing – review and editing, funding acquisition. **Christian Bock:** conceptualisation, writing – review and editing, funding acquisition.

## Funding

This study was supported partially by grants from the DFG (DR298/13‐1 DR 298/13‐2, BO2467/4‐1, BO2467/4‐2 and WE6575/1‐1).

## Conflicts of Interest

The authors declare no conflicts of interest.

## Supporting information


**Table S1:** Fitted model functions δ (pH, T) for the 15 examined metabolic compounds $\left(∆,T,=,\frac{T-T_{\mathrm{ref}}}{K},;,T_{\mathrm{ref}},=,273.15,K\right)$. The last two columns summarise the effects of temperature (T) and pH on the chemical shift of the 15 metabolites. The arrows indicate the direction of the change (← down‐field, → up‐field). The green fields mark signals whose difference in chemical shift between the lowest and highest temperatures is smaller than the average difference across all signals examined, while the red fields mark resonances with a higher difference. The same applies to the difference in chemical shifts between the alkaline and basic pH values. It is important to note that the up‐field and down‐field signals were considered separately.
**Table S2:** Fitted model functions J (pH, T) for the examined metabolic compounds $\left(∆,T,=,\frac{T-T_{\mathrm{ref}}}{K},;,T_{\mathrm{ref}},=,273.15,K\right)$. It is essential to note that this study's evaluation is based on peak picking and multiplet analysis, which is why only the J‐coupling constants that can be determined based on this simplification are presented. The last two columns summarise the effects of temperature (T) and pH on the J‐coupling constants. The arrows indicate the direction of the change (↑ greater, ↓ lower). The green fields mark signals whose difference in J‐coupling constant between the lowest and highest temperatures is smaller than the average difference across all signals examined, while the red fields mark constants with a higher difference. The same applies to the difference in J‐coupling constants between the alkaline and basic pH values.
**Figure S1:** Spectra of N‐acetylaspartate (NAA) referenced to DSS (pH 7, 40°C) (A) and example spectra for the pH dependence of the resonances in the upfield region (B) and the temperature dependence of the NH resonance (C). Experimentally determined ^1^H chemical shifts (δ [ppm]) and J‐coupling constants (J[Hz]) and the fitted model functions (δ (pH, T), J (pH, T)) for the signals of NAA (D–K).
**Figure S2:** Spectra of alanine (Ala) referenced to DSS (pH 7, 40°C) (A) and example spectra for the pH (B) and the temperature (C) dependence of the resonances. Experimentally determined ^1^H chemical shifts (δ [ppm]) and J‐coupling constants (J[Hz]) and the fitted model functions (δ (pH, T), J (pH, T)) for the signals of Ala (D‐F).
**Figure S3:** Spectra of γ‐Aminobutyric acid (GABA) referenced to DSS (pH 7, 40°C) (A) and example spectra for the temperature dependence of the resonances (B). Experimentally determined ^1^H chemical shifts (δ [ppm]) and J‐coupling constants (J[Hz]) and the fitted model functions (δ (pH, T), J (pH, T)) for the signals of GABA (C–F).
**Figure S4:** Spectra of aspartate (Asp) referenced to DSS (pH 7, 40°C) (A) and example spectra for the pH (B) and temperature (C) dependence of the resonances. Experimentally determined ^1^H chemical shifts (δ [ppm]) and J‐coupling constants (J[Hz]) and the fitted model functions (δ (pH, T), J (pH, T)) for the signals of Asp (D–I).
**Figure S5:** Spectra of choline (Cho) referenced to DSS (pH 7, 40°C) (A) and example spectra for the temperature dependence of the resonances (B). Experimentally determined ^1^H chemical shifts (δ [ppm]) and the fitted model functions (δ (pH, T)) for the signals of Cho (C–E).
**Figure S6:** Spectra of creatine (Cr) referenced to DSS (pH 7, 40°C) (A) and example spectra for the temperature dependence of the resonances (B) and the pH dependence of the NH resonance (C). Experimentally determined ^1^H chemical shifts (δ [ppm]) and the fitted model functions (δ (pH, T)) for the signals of Cr (D–F).
**Figure S7:** Spectra of glutamate (Glu) referenced to DSS (pH 7, 40°C) (A) and example spectra for the temperature dependence of the resonances (B). Experimentally determined ^1^H chemical shifts (δ [ppm]) and the fitted model functions (δ (pH, T)) for the signals of Glu (C–G).
**Figure S8:** Spectra of glutamine (Gln) referenced to DSS (pH 7, 40°C) (A) and example spectra for the pH dependence of the resonances in the upfield region (B) and the temperature dependence of the NH resonances (C). Experimentally determined ^1^H chemical shifts (δ [ppm]) and the fitted model functions (δ (pH, T)) for the signals of Gln (D–J).
**Figure S9:** Spectra of glycine (Gly) referenced to DSS (pH 7, 40°C) (A) and example spectra for the temperature dependence of the singlet (B). Experimentally determined ^1^H chemical shifts (δ [ppm]) and the fitted model function (δ (pH, T)) for the singlet of Gly (C).
**Figure S10:** Spectra of histidine (His) referenced to DSS (pH 7, 40°C) (A) and example spectra for the pH dependence of the resonances in the upfield region (B) and of the NH resonances (C). Experimentally determined ^1^H chemical shifts (δ [ppm]) and J‐coupling constants (J[Hz]) and the fitted model functions (δ (pH, T), J (pH, T)) for the signals of His (D‐K).
**Figure S11:** Spectra of myo‐inositol (m‐Ins) referenced to DSS (pH 7, 40°C) (A) and example spectra for the pH dependence of the resonances (B). Experimentally determined ^1^H chemical shifts (δ [ppm]) and the fitted model functions (δ (pH, T)) for the signals of m‐Ins (C–F).
**Figure S12:** Spectra of lactate (Lac) referenced to DSS (pH 7, 40°C) (A) and example spectra for the temperature dependence of the resonances (B). Experimentally determined ^1^H chemical shifts (δ [ppm]) and J‐coupling constant (J[Hz]) and the fitted model functions (δ (pH, T), J (pH, T)) for the signals of Lac (C–E).
**Figure S13:** Spectra of phosphocreatine (PCr) referenced to DSS (pH 7, 40°C) (A) and example spectra for the temperature dependence of the resonances in the upfield region (B) and of the NH resonances (C). Experimentally determined ^1^H chemical shifts (δ [ppm]) and the fitted model functions (δ (pH, T)) for the signals of PCr (D–G).
**Figure S14:** Spectra of taurine (Tau) referenced to DSS (pH 7, 40°C) (A) and example spectra for the pH dependence of the resonances (B). Experimentally determined ^1^H chemical shifts (δ [ppm]) and the fitted model functions (δ (pH, T)) for the signals of Tau (C, D).
**Figure S15:** Spectra of threonine (Thr) referenced to DSS (pH 7, 40°C) (A) and example spectra for the pH dependence of the resonances. Experimentally determined ^1^H chemical shifts (δ [ppm]) and J‐coupling constants (J[Hz]) and the fitted model functions (δ (pH, T), J (pH, T)) for the signals of Thr (C–G).
**Figure S16:** Metabolite concentrations determined by the AQSES algorithm for the examined temperature and pH range. The results are given as a percentage of the simulated values.
**Figure S17:** nbm70239‐sup‐0001‐Supplementary_Material.docx. ^1^H NMR spectrum simulated for pH 7.2 and 32°C and the resulting residual spectrum using the AQSES algorithm, with chemical shift values and J‐coupling constants for 37°C and pH 7 as prior knowledge (A). Subplots (B), (C) and (D) show the spectra for (pH 7.2 and 35°C, 37°C and 40°C), respectively and also the corresponding residual spectra.
**Figure S18:** nbm70239‐sup‐0001‐Supplementary_Material.docx. ^1^H NMR spectrum simulated for pH 6.5 and 37°C and the resulting residual spectrum using the AQSES algorithm, with chemical shift values and J‐coupling constants for 37°C and pH 7 as prior knowledge (A). Subplots (B) and (C) show the spectra for (pH 7.2 and pH 7.7, 37°C), respectively and also the corresponding residual spectra.

## Data Availability

The data supporting this study's findings are available from the corresponding author upon reasonable request. After approval, the data will be made available in a public repository.
