# Supplementary material for: ¹H NMR Chemical Shifts and J‐Coupling Constants Dependence on Temperature and pH: Implications for the Quantification of Relevant Metabolites
Source: NMR Biomed. 2026 Feb 16;39(3):e70239. doi: 10.1002/nbm.70239 (PMC12910193; doi:10.1002/nbm.70239)
Supplement: Supplementary file 1 — Table S1: Fitted model functions δ (pH, T) for the 15 examined metabolic compounds ∆T=T−TrefK;Tref=273.15K. The last two columns summarise the effects of temperature (T) and pH on the chemical shift of the 15 metabolites. The arrows indicate the direction of the change (← down‐field, → up‐field). The green fields mark signals whose difference in chemical shift between the lowest and highest temperatures is smaller than the average difference across all signals examined, while the red fields mark resonances with a higher difference. The same applies to the difference in chemical shifts between the alkaline and basic pH values. It is important to note that the up‐field and down‐field signals were considered separately. Table S2: Fitted model functions J (pH, T) for the examined metabolic compounds ∆T=T−TrefK;Tref=273.15K. It is essential to note that this study's evaluation is based on peak picking and multiplet analysis, which is why only the J‐coupling constants that can be determined based on this simplification are presented. The last two columns summarise the effects of temperature (T) and pH on the J‐coupling constants. The arrows indicate the direction of the change (↑ greater, ↓ lower). The green fields mark signals whose difference in J‐coupling constant between the lowest and highest temperatures is smaller than the average difference across all signals examined, while the red fields mark constants with a higher difference. The same applies to the difference in J‐coupling constants between the alkaline and basic pH values. Figure S1: Spectra of N‐acetylaspartate (NAA) referenced to DSS (pH 7, 40°C) (A) and example spectra for the pH dependence of the resonances in the upfield region (B) and the temperature dependence of the NH resonance (C). Experimentally determined 1H chemical shifts (δ [ppm]) and J‐coupling constants (J[Hz]) and the fitted model functions (δ (pH, T), J (pH, T)) for the signals of NAA (D–K). Figure S2: Spectra of alanine (Ala) referenced t [file NBM-39-e70239-s001.docx]

^1^H NMR chemical shifts and J-coupling constants dependence on temperature and pH: Implications for the quantification of relevant metabolites

Felizitas C. Wermter, Christian Bock, Wolfgang Dreher

Table S 1: Fitted model functions δ(pH, T) for the 15 examined metabolic compounds ($\Delta T=\frac{T-T_{ref}}{K}; T_{ref}=273.15K)$. The last two columns summarise the effects of temperature (T) and pH on the chemical shift of the 15 metabolites. The arrows indicate the direction of the change (🡨 down-field, 🡪 up-field). The green fields mark signals whose difference in chemical shift between the lowest and highest temperatures is smaller than the average difference across all signals examined, while the red fields mark resonances with a higher difference. The same applies to the difference in chemical shifts between the alkaline and basic pH values. It is important to note that the up-field and down-field signals were considered separately.

| Compound/ Group | |  | $a(T)$ | $b(T)$ | $c(T)$ | T ↑ | pH ↑ |
| --- | --- | --- | --- | --- | --- | --- | --- |
| NAA | ^2^CH_3_ | $\delta\left( pH,T \right)=a\left( T \right)+\left( \frac{b\left( T \right)-a\left( T \right)}{1+{10}^{\left( c\left( T \right)-pH \right)}} \right)$ | $3.0586+9.99*{10}^{-3}\Delta T-0.10*{10}^{-3}{\Delta T}^{2}$ | $2.0073+1.01*{10}^{-4}\Delta T-0.25*{10}^{-5}{\Delta T}^{2}$ | $2.5957-4.34*{10}^{-4}\Delta T+0.26*{10}^{-4}{\Delta T}^{2}$ | 🡪 | 🡪 |
|  | ^2^CH |  | $5.1163+9.99*{10}^{-3}\Delta T+0.10*{10}^{-3}{\Delta T}^{2}$ | $4.3750+1.51*{10}^{-4}\Delta T-0.10*{10}^{-5}{\Delta T}^{2}$ | $3.7321-1.29*{10}^{-3}\Delta T+0.10*{10}^{-3}{\Delta T}^{2}$ | 🡨 | 🡪 |
|  | ^3^CH_2_ |  | $2.9737-4.60*{10}^{-3}\Delta T+0.67*{10}^{-7}{\Delta T}^{2}$ | $2.6948-5.99*{10}^{-4}\Delta T-0.12*{10}^{-5}{\Delta T}^{2}$ | $4.2078+1.11*{10}^{-2}\Delta T+0.99*{10}^{-4}{\Delta T}^{2}$ | 🡪 | 🡪 |
|  | ^3’^CH_2_ |  | $2.9136-2.32*{10}^{-3}\Delta T+0.12*{10}^{-4}{\Delta T}^{2}$ | $2.4831+5.43*{10}^{-5}\Delta T-0.14*{10}^{-5}{\Delta T}^{2}$ | $4.2968+6.25*{10}^{-3}\Delta T-0.45*{10}^{-4}{\Delta T}^{2}$ | 🡪 | 🡪 |
|  | NH |  | $8.1470-7.83*{10}^{-3}\Delta T-0.19*{10}^{-4}{\Delta T}^{2}$ | $8.1414-1.00{*10}^{-2}\Delta T+0.33*{10}^{-4}{\Delta T}^{2}$ | $6.8615-4.99*{10}^{-2}\Delta T+0.99*{10}^{-4}{\Delta T}^{2}$ | 🡪 | 🡪 |
| Ala | ^2^CH |  | $3.7665+5.17*{10}^{-4}\Delta T-0.50*{10}^{-5}{\Delta T}^{2}$ | $3.1772+1.00{*10}^{-2}\Delta T-0.96*{10}^{-4}\Delta T^{2}$ | $10.2462-1.90*{10}^{-2}\Delta T+0.88*{10}^{-4}{\Delta T}^{2}$ | 🡨 | 🡪 |
|  | ^3^CH_3_ |  | $1.4646+1.23*{10}^{-4}\Delta T-0.80*{10}^{-6}{\Delta T}^{2}$ | $-0.6989-0.99*{10}^{-2}\Delta T-0.10*{10}^{-3}\Delta T^{2}$ | $11.0983-1.10*{10}^{-2}\Delta T+0.98*{10}^{-4}{\Delta T}^{2}$ | 🡨 | 🡪 |
| GABA | ^2^CH_2_ |  | $2.4071+2.84*{10}^{-3}\Delta T-0.51*{10}^{-4}{\Delta T}^{2}$ | $2.2857-2.50*{10}^{-5}\Delta T-0.20*{10}^{-6}{\Delta T}^{2}$ | $4.3827-4.11*{10}^{-3}\Delta T-0.98*{10}^{-4}{\Delta T}^{2}$ | 🡪 | 🡪 |
|  | ^3^CH_2_ |  | $2.6852-4.56*{10}^{-3}\Delta T-0.10*{10}^{-3}{\Delta T}^{2}$ | $1.8891-5.83*{10}^{-5}\Delta T+0.50*{10}^{-6}{\Delta T}^{2}$ | $2.9404+7.89*{10}^{-3}\Delta T+0.99*{10}^{-4}{\Delta T}^{2}$ | 🡪 | 🡪 |
|  | ^4^CH_2_ |  | $4.1162-9.28*{10}^{-3}\Delta T-0.10*{10}^{-3}{\Delta T}^{2}$ | $2.9870-8.10*{10}^{-4}\Delta T-0.17*{10}^{-5}{\Delta T}^{2}$ | $2.9146-7.43*{10}^{-5}\Delta T-0.99*{10}^{-4}{\Delta T}^{2}$ | 🡪 | 🡪 |
| Asp | ^2^CH |  | $3.8900+2.54*{10}^{-4}\Delta T-0.29*{10}^{-5}{\Delta T}^{2}$ | $3.8820+4.96*{10}^{-4}\Delta T-0.14*{10}^{-4}{\Delta T}^{2}$ | $6.3401+3.35*{10}^{-2}\Delta T+0.17*{10}^{-3}{\Delta T}^{2}$ | 🡨 | 🡪 |
|  | ^3^CH_2_ |  | $2.9935+9.31*{10}^{-3}\Delta T-0.16*{10}^{-4}{\Delta T}^{2}$ | $2.8016+1.01*{10}^{-5}\Delta T-0.22*{10}^{-5}{\Delta T}^{2}$ | $3.7487-8.92*{10}^{-3}\Delta T+0.96*{10}^{-4}{\Delta T}^{2}$ | 🡪 | 🡪 |
|  | ^3’^CH_2_ |  | $2.6789-4.30*{10}^{-4}\Delta T-0.24*{10}^{-5}{\Delta T}^{2}$ | $2.2320-9.39*{10}^{-3}\Delta T-0.10*{10}^{-3}{\Delta T}^{2}$ | $9.8874-9.35*{10}^{-4}\Delta T-0.82*{10}^{-4}{\Delta T}^{2}$ | 🡪 | 🡪 |
| Cho | N(CH_3_)_3_ |  | $3.2023-5.27*{10}^{-4}\Delta T+0.25*{10}^{-5}{\Delta T}^{2}$ | $9.9773+9.90*{10}^{-3}\Delta T-0.82*{10}^{-4}{\Delta T}^{2}$ | $23.2103+9.99*{10}^{-1}\Delta T+0.90*{10}^{-4}{\Delta T}^{2}$ | 🡪 | = |
|  | ^1^CH_2_ |  | $4.0596-1.82*{10}^{-4}\Delta T+0.08*{10}^{-5}{\Delta T}^{2}$ | $1.9001-1.53*{10}^{-2}\Delta T-0.49*{10}^{-3}{\Delta T}^{2}$ | $21.3854+9.93*{10}^{-1}\Delta T+0.18*{10}^{-3}{\Delta T}^{2}$ | 🡪 | = |
|  | ^2^CH_2_ |  | $3.5249-7.14*{10}^{-4}\Delta T+0.33*{10}^{-5}{\Delta T}^{2}$ | $7.9917-9.97*{10}^{-3}\Delta T-0.99*{10}^{-4}{\Delta T}^{2}$ | $18.3074+9.73*{10}^{-1}\Delta T+0.96*{10}^{-4}{\Delta T}^{2}$ | 🡪 | = |
| Cr | N(CH^3^) |  | $3.0192+2.67*{10}^{-4}\Delta T-0.10*{10}^{-5}{\Delta T}^{2}$ | $3.0187+2.92*{10}^{-4}\Delta T-0.16*{10}^{-5}{\Delta T}^{2}$ | $6.7217+0.80*{10}^{-2}\Delta T+0.99*{10}^{-4}{\Delta T}^{2}$ | 🡨 | 🡪 |
|  | ^2^CH_2_ |  | $4.6019+9.99*{10}^{-3}\Delta T-0.46*{10}^{-4}\Delta T^{2}$ | $3.9294-3.02*{10}^{-4}\Delta T-0.25*{10}^{-5}{\Delta T}^{2}$ | $2.5139-3.66*{10}^{-3}\Delta T+0.10*{10}^{-3}{\Delta T}^{2}$ | 🡪 | 🡪 |
|  | NH |  | $6.8068-4.36*{10}^{-3}\Delta T-0.37*{10}^{-4}\Delta T^{2}$ | $6.8102-4.60*{10}^{-3}\Delta T-0.06*{10}^{-4}\Delta T^{2}$ | $6.4204-11.79*{10}^{-2}\Delta T+0.52*{10}^{-4}\Delta T^{2}$ | 🡪 | = |
| Glu | ^2^CH |  | $3.7541-3.70*{10}^{-5}\Delta T-0.24*{10}^{-5}\Delta T^{2}$ | $3.5690+7.60*{10}^{-3}\Delta T-0.99*{10}^{-4}\Delta T^{2}$ | $9.4574-3.07*{10}^{-2}\Delta T+0.99*{10}^{-4}\Delta T^{2}$ | 🡪 | 🡪 |
|  | ^3^CH_2_ |  | $2.0548-3.75*{10}^{-4}\Delta T+0.80*{10}^{-6}\Delta T^{2}$ | $2.0491-4.06*{10}^{-4}\Delta T-0.14*{10}^{-5}\Delta T^{2}$ | $7.2924+1.51*{10}^{-2}\Delta T-0.10*{10}^{-3}\Delta T^{2}$ | 🡪 | 🡪 |
|  | ^3’^CH_2_ |  | $2.1133+2.48*{10}^{-4}\Delta T+0.97*{10}^{-7}\Delta T^{2}$ | $2.1060+3.64*{10}^{-4}\Delta T-0.42*{10}^{-5}\Delta T^{2}$ | $7.7460-1.17*{10}^{-3}\Delta T+0.99*{10}^{-4}\Delta T^{2}$ | 🡨 | 🡪 |
|  | ^4^CH_2_ |  | $2.3188+7.22*{10}^{-4}\Delta T-0.60*{10}^{-6}\Delta T^{2}$ | $2.2991+1.47*{10}^{-3}\Delta T-0.12*{10}^{-4}\Delta T^{2}$ | $9.4593-7.72*{10}^{-2}\Delta T+0.98*{10}^{-4}\Delta T^{2}$ | 🡨 | 🡪 |
|  | ^4’^CH_2_ |  | $2.3485+1.81*{10}^{-4}\Delta T+0.46*{10}^{-5}\Delta T^{2}$ | $2.3418+3.43*{10}^{-4}\Delta T-0.33*{10}^{-5}\Delta T^{2}$ | $7.4598-3.32*{10}^{-2}\Delta T+0.99*{10}^{-4}\Delta T^{2}$ | 🡨 | 🡨 |

| Compound/ Group | |  | $a(T)$ | $b(T)$ | $c(T)$ | T ↑ | pH ↑ |
| --- | --- | --- | --- | --- | --- | --- | --- |
| Gln | ^2^CH | $\delta\left( pH,T \right)=a\left( T \right)+\left( \frac{b\left( T \right)-a\left( T \right)}{1+{10}^{\left( c\left( T \right)-pH \right)}} \right)$ | $3.7715+5.73*{10}^{-5}\Delta T-0.40*{10}^{-5}\Delta T^{2}$ | $3.5961+4.98*{10}^{-3}\Delta T-0.65*{10}^{-4}\Delta T^{2}$ | $8.9758-2.96*{10}^{-2}\Delta T+0.98*{10}^{-4}\Delta T^{2}$ | 🡪 | 🡪 |
|  | ^3^CH_2_ |  | $2.1309+2.16*{10}^{-4}\Delta T-0.40*{10}^{-6}\Delta T^{2}$ | $1.2922+9.99*{10}^{-3}\Delta T-0.99*{10}^{-4}\Delta T^{2}$ | $10.0053-1.64*{10}^{-2}\Delta T+0.80*{10}^{-4}\Delta T^{2}$ | 🡨 | 🡪 |
|  | ^3’^CH_2_ |  | $2.1225-1.26*{10}^{-4}\Delta T-0.13*{10}^{-3}\Delta T^{2}$ | $1.2360+7.53*{10}^{-3}\Delta T-0.10*{10}^{-3}\Delta T^{2}$ | $9.9137-1.52*{10}^{-2}\Delta T+0.99*{10}^{-4}\Delta T^{2}$ | 🡪 | 🡪 |
|  | ^4^CH_2_ |  | $2.4159+7.04*{10}^{-4}\Delta T-0.37*{10}^{-5}\Delta T^{2}$ | $2.2399-3.15*{10}^{-3}\Delta T-0.99*{10}^{-4}\Delta T^{2}$ | $9.5395-1.78*{10}^{-3}\Delta T+0.95*{10}^{-4}\Delta T^{2}$ | 🡨 | 🡪 |
|  | ^4’^CH_2_ |  | $2.4515+2.89*{10}^{-4}\Delta T-0.22*{10}^{-5}\Delta T^{2}$ | $1.5081-9.97*{10}^{-3}\Delta T-0.10*{10}^{-3}\Delta T^{2}$ | $10.2591-8.43*{10}^{-3}\Delta T+0.97*{10}^{-4}\Delta T^{2}$ | 🡨 | 🡪 |
|  | NH_2_ |  | $7.0195-6.78*{10}^{-3}\Delta T+0.53*{10}^{-4}{\Delta T}^{2}$ | $7.0122-5.91*{10}^{-3}\Delta T+0.86*{10}^{-5}{\Delta T}^{2}$ | $6.2852-3.90*{10}^{-2}\Delta T+0.43*{10}^{-4}{\Delta T}^{2}$ | 🡪 | 🡪 |
|  |  |  | $7.7693-7.29*{10}^{-3}\Delta T+0.73*{10}^{-3}{\Delta T}^{2}$ | $7.8747-1.00*{10}^{-2}\Delta T-0.14*{10}^{-4}{\Delta T}^{2}$ | $9.9758-4.82*{10}^{-2}\Delta T+0.55*{10}^{-4}{\Delta T}^{2}$ | 🡪 | 🡪 |
| Gly | ^2^CH_2_ |  | $3.5474+2.96*{10}^{-4}\Delta T-0.44*{10}^{-5}{\Delta T}^{2}$ | $3.2996-9.81*{10}^{-3}\Delta T+0.10*{10}^{-3}{\Delta T}^{2}$ | $10.2444-1.83*{10}^{-2}\Delta T-0.98*{10}^{-4}{\Delta T}^{2}$ | 🡪 | 🡪 |
| His | ^α^CH |  | $4.0210+7.85*{10}^{-5}\Delta T-0.40*{10}^{-5}{\Delta T}^{2}$ | $3.9552+2.61*{10}^{-4}\Delta T-0.95*{10}^{-5}{\Delta T}^{2}$ | $6.7475-2.45*{10}^{-3}\Delta T+0.99*{10}^{-4}{\Delta T}^{2}$ | 🡪 | 🡪 |
|  | ^β^CH_2_ |  | $3.2951+3.52*{10}^{-4}\Delta T-0.12*{10}^{-4}{\Delta T}^{2}$ | $3.0749+8.39*{10}^{-4}\Delta T-0.13*{10}^{-4}{\Delta T}^{2}$ | $6.5393-8.86*{10}^{-3}\Delta T+0.99*{10}^{-4}{\Delta T}^{2}$ | 🡪 | 🡪 |
|  | ^β’^CH_2_ |  | $3.3448+6.22*{10}^{-5}\Delta T-0.60*{10}^{-5}{\Delta T}^{2}$ | $3.2001+3.26*{10}^{-4}\Delta T-0.41*{10}^{-5}{\Delta T}^{2}$ | $6.5669-8.26*{10}^{-3}\Delta T+0.99*{10}^{-4}{\Delta T}^{2}$ | 🡪 | 🡪 |
|  | ^2^CH* |  | $8.6571-3.41*{10}^{-4}\Delta T-0.18*{10}^{-4}{\Delta T}^{2}$ | $7.7149+1.40*{10}^{-3}\Delta T-0.29*{10}^{-4}{\Delta T}^{2}$ | $6.3729-7.41*{10}^{-3}\Delta T+0.51*{10}^{-4}{\Delta T}^{2}$ | 🡪 | 🡪 |
|  | ^5^CH* |  | $7.3581+3.61*{10}^{-3}\Delta T-0.90*{10}^{-4}{\Delta T}^{2}$ | $7.0244+1.13*{10}^{-4}\Delta T-0.63*{10}^{-5}{\Delta T}^{2}$ | $6.3706-8.84*{10}^{-3}\Delta T+0.10*{10}^{-4}{\Delta T}^{2}$ | 🡪 | 🡪 |
| m-Ins | ^1-3^CH |  | $5.8053-9.47*{10}^{-3}\Delta T-0.21*{10}^{-4}{\Delta T}^{2}$ | $3.5262+2.88*{10}^{-5}\Delta T-0.21*{10}^{-5}{\Delta T}^{2}$ | $2.3254+1.41*{10}^{-2}\Delta T-0.10*{10}^{-4}{\Delta T}^{2}$ | 🡪 | 🡪 |
|  | ^2^CH |  | $4.2030+1.00*{10}^{-2}\Delta T-0.88*{10}^{-4}{\Delta T}^{2}$ | $4.0444+4.33*{10}^{-4}\Delta T-0.23*{10}^{-5}{\Delta T}^{2}$ | $3.4353-5.02*{10}^{-3}\Delta T+0.99*{10}^{-4}{\Delta T}^{2}$ | 🡨 | 🡪 |
|  | ^4-6^CH |  | $3.7059+8.10*{10}^{-3}\Delta T+0.10*{10}^{-3}{\Delta T}^{2}$ | $3.6068+2.70*{10}^{-4}\Delta T+0.40*{10}^{-6}{\Delta T}^{2}$ | $3.6497-1.42*{10}^{-2}\Delta T+0.99*{10}^{-4}{\Delta T}^{2}$ | 🡨 | 🡪 |
|  | ^5^CH |  | $4.6053+9.78*{10}^{-3}\Delta T-0.10*{10}^{-4}{\Delta T}^{2}$ | $3.2611+4.36*{10}^{-4}\Delta T-0.32*{10}^{-5}{\Delta T}^{2}$ | $2.5993+1.77*{10}^{-3}\Delta T+0.97*{10}^{-4}{\Delta T}^{2}$ | 🡨 | 🡪 |
| Lac | ^2^CH |  | $4.1476+1.40*{10}^{-3}\Delta T+0.80*{10}^{-6}{\Delta T}^{2}$ | $4.1143-3.37*{10}^{-4}\Delta T-0.29*{10}^{-5}{\Delta T}^{2}$ | $4.5772-1.39*{10}^{-2}\Delta T+0.99*{10}^{-4}{\Delta T}^{2}$ | 🡪 | 🡪 |
|  | ^3^CH_3_ |  | $1.3932-3.30*{10}^{-3}\Delta T+0.37*{10}^{-4}{\Delta T}^{2}$ | $1.3196-1.26*{10}^{-4}\Delta T-0.80*{10}^{-6}{\Delta T}^{2}$ | $3.7786+2.75*{10}^{-2}\Delta T+0.97*{10}^{-4}{\Delta T}^{2}$ | 🡪 | 🡪 |
| PCr | N(CH_3_) |  | $3.0461+7.61*{10}^{-4}\Delta T+0.10*{10}^{-3}{\Delta T}^{2}$ | $3.0301+4.02*{10}^{-5}\Delta T-0.16*{10}^{-5}{\Delta T}^{2}$ | $4.6511-2.81*{10}^{-2}\Delta T+0.99*{10}^{-4}{\Delta T}^{2}$ | 🡪 | 🡪 |
|  | ^2^CH_2_ |  | $3.9682+1.64*{10}^{-4}\Delta T+0.11*{10}^{-4}{\Delta T}^{2}$ | $3.9557-5.97*{10}^{-4}\Delta T-0.25*{10}^{-5}{\Delta T}^{2}$ | $4.8312-1.67*{10}^{-2}\Delta T+0.99*{10}^{-4}{\Delta T}^{2}$ | 🡪 | 🡪 |
|  | NH |  | $6.7877+1.05*{10}^{-3}\Delta T-0.10*{10}^{-3}{\Delta T}^{2}$ | $6.8200-9.56*{10}^{-3}\Delta T+0.99*{10}^{-4}{\Delta T}^{2}$ | $5.5155+6.16*{10}^{-4}\Delta T+0.10*{10}^{-3}{\Delta T}^{2}$ | 🡪 | 🡪 |
|  | NH |  | $9.9266-9.92*{10}^{-3}\Delta T-0.96*{10}^{-4}{\Delta T}^{2}$ | $7.3899-1.58*{10}^{-4}\Delta T-0.70*{10}^{-4}{\Delta T}^{2}$ | $2.1434+2.14*{10}^{-2}\Delta T+0.10*{10}^{-3}{\Delta T}^{2}$ | 🡪 | 🡪 |
| Tau | ^1^CH_2_ |  | $3.4121+4.67*{10}^{-4}\Delta T-0.21*{10}^{-5}{\Delta T}^{2}$ | $3.2135+6.83*{10}^{-3}\Delta T-0.83*{10}^{-4}{\Delta T}^{2}$ | $8.9397-2.88*{10}^{-2}\Delta T+0.94*{10}^{-4}{\Delta T}^{2}$ | 🡪 | 🡪 |
|  | ^2^CH_2_ |  | $3.2517+6.06*{10}^{-5}\Delta T-0.35*{10}^{-5}{\Delta T}^{2}$ | $3.0972+5.25*{10}^{-3}\Delta T-0.61*{10}^{-4}{\Delta T}^{2}$ | $9.1133-2.51*{10}^{-2}\Delta T-0.10*{10}^{-3}{\Delta T}^{2}$ | 🡪 | 🡪 |
| Thr | ^2^CH |  | $3.5741+6.59*{10}^{-4}\Delta T-0.67*{10}^{-5}{\Delta T}^{2}$ | $3.4744+2.34*{10}^{-3}\Delta T-0.31*{10}^{-4}{\Delta T}^{2}$ | $8.5813-1.76*{10}^{-2}\Delta T+0.99*{10}^{-4}{\Delta T}^{2}$ | = | 🡪 |
|  | ^3^CH |  | $4.2452+4.00*{10}^{-4}\Delta T-0.33*{10}^{-5}{\Delta T}^{2}$ | $4.2249+2.20*{10}^{-3}\Delta T-0.10*{10}^{-3}{\Delta T}^{2}$ | $7.3001+3.23*{10}^{-2}\Delta T-0.97*{10}^{-4}{\Delta T}^{2}$ | 🡨 | 🡪 |
|  | ^4^CH_3_ |  | $1.3156+1.21*{10}^{-4}\Delta T-0.90*{10}^{-6}{\Delta T}^{2}$ | $1.2873+4.86*{10}^{-4}\Delta T-0.49*{10}^{-5}{\Delta T}^{2}$ | $8.6512-1.33*{10}^{-2}\Delta T-0.12*{10}^{-5}{\Delta T}^{2}$ | = | 🡪 |

Table S 2: Fitted model functions J(pH, T) for the examined metabolic compounds ($\Delta T=\frac{T-T_{ref}}{K}; T_{ref}=273.15K)$. It is essential to note that this study's evaluation is based on peak picking and multiplet analysis, which is why only the J-coupling constants that can be determined based on this simplification are presented. The last two columns summarise the effects of temperature (T) and pH on the J-coupling constants. The arrows indicate the direction of the change (↑ greater, ↓ lower). The green fields mark signals whose difference in J-coupling constant between the lowest and highest temperatures is smaller than the average difference across all signals examined, while the red fields mark constants with a higher difference. The same applies to the difference in J-coupling constants between the alkaline and basic pH values.

|  |  |  |  |  |  | T ↑ | pH ↑ |
| --- | --- | --- | --- | --- | --- | --- | --- |
| NAA | 2-3 | $J\left( pH,T \right)=a(T)+(\frac{b\left( T \right)-a\left( T \right)}{1+{10}^{\left( c\left( T \right)-pH \right)}})$ | $5.05+9.41*{10}^{-3}\Delta T-0.83*{10}^{-4}{\Delta T}^{2}$ | $3.59+1.00*{10}^{-2}\Delta T-0.91*{10}^{-5}{\Delta T}^{2}$ | $4.38+1.01*{10}^{-2}\Delta T-0.98*{10}^{-4}{\Delta T}^{2}$ | ↑ | ↓ |
|  | 2-3‘ |  | $9.12-1.98*{10}^{-2}\Delta T+0.20*{10}^{-3}{\Delta T}^{2}$ | $10.39-1.99*{10}^{-2}\Delta T+0.45*{10}^{-4}{\Delta T}^{2}$ | $4.89-1.62*{10}^{-3}\Delta T+0.19*{10}^{-3}{\Delta T}^{2}$ | ↓ | ↑ |
|  | 3-3‘ |  | $-16.35-6.84*{10}^{-3}\Delta T+0.15*{10}^{-3}{\Delta T}^{2}$ | $-15.86+8.08*{10}^{-3}\Delta T-0.13*{10}^{-4}{\Delta T}^{2}$ | $4.78-9.74*{10}^{-3}\Delta T+0.14*{10}^{-3}{\Delta T}^{2}$ | ↑ | ↑ |
| GABA | 2-3, 2-3‘, 2‘-3, 2‘-3‘ |  | $7.41-8.09*{10}^{-4}\Delta T-0.30*{10}^{-4}{\Delta T}^{2}$ | $4.91-1.93*{10}^{-2}\Delta T+0.20*{10}^{-3}{\Delta T}^{2}$ | $22.78-1.21*{10}^{-1}\Delta T+0.86*{10}^{-7}{\Delta T}^{2}$ | ↓ | = |
| Asp | 2-3 |  | $4.89-1.76*{10}^{-2}\Delta T+0.20*{10}^{-3}{\Delta T}^{2}$ | $3.75+2.67*{10}^{-3}\Delta T-0.11*{10}^{-4}{\Delta T}^{2}$ | $3.65-5.99*{10}^{-3}\Delta T+0.19*{10}^{-3}{\Delta T}^{2}$ | ↑ | ↑ |
|  | 2-3‘ |  | $8.49+1.05*{10}^{-3}\Delta T-0.60*{10}^{-5}{\Delta T}^{2}$ | $8.52+5.77*{10}^{-3}\Delta T-0.15*{10}^{-4}{\Delta T}^{2}$ | $5.66-2.33*{10}^{-2}\Delta T+0.94*{10}^{-4}{\Delta T}^{2}$ | ↑ | ↑ |
|  | 3-3‘ |  | $-17.70+1.16*{10}^{-2}\Delta T-0.11*{10}^{-3}{\Delta T}^{2}$ | $-17.57+5.12*{10}^{-3}\Delta T+0.52*{10}^{-5}{\Delta T}^{2}$ | $4.71+6.31*{10}^{-2}\Delta T+0.11*{10}^{-3}{\Delta T}^{2}$ | ↑ | ↑ |
| His | $\alpha$-$\beta$ |  | $7.14-1.51*{10}^{-2}\Delta T+0.49*{10}^{-4}{\Delta T}^{2}$ | $8.30-1.29*{10}^{-2}\Delta T+0.10*{10}^{-3}{\Delta T}^{2}$ | $6.47-1.30*{10}^{-2}\Delta T+0.62*{10}^{-4}{\Delta T}^{2}$ | ↓ | ↑ |
|  | $\alpha$-$\beta$‘ |  | $5.94+1.84*{10}^{-2}\Delta T-0.16*{10}^{-3}{\Delta T}^{2}$ | $4.59+7.76*{10}^{-3}\Delta T-0.70*{10}^{-3}{\Delta T}^{2}$ | $6.41-1.13*{10}^{-2}\Delta T+0.47*{10}^{-4}{\Delta T}^{2}$ | ↓ | ↓ |
|  | $\beta$-$\beta$‘ |  | $-15.75-6.08*{10}^{-3}\Delta T+1.11*{10}^{-4}{\Delta T}^{2}$ | $-15.35-3.91*{10}^{-3}\Delta T+0.26*{10}^{-4}{\Delta T}^{2}$ | $6.65-2.01*{10}^{-2}\Delta T+0.35*{10}^{-3}{\Delta T}^{2}$ | ↓ | ↑ |
| Lac | 2-3 |  | $6.95-9.06*{10}^{-4}\Delta T-0.34*{10}^{-5}{\Delta T}^{2}$ | $6.93-9.09*{10}^{-4}\Delta T-0.41*{10}^{-4}{\Delta T}^{2}$ | $8.55+5.51*{10}^{-2}\Delta T+0.40*{10}^{-3}{\Delta T}^{2}$ | ↓ | = |
| Thr | 2-3 |  | $4.85-3.90*{10}^{-3}\Delta T-0.21*{10}^{-5}{\Delta T}^{2}$ | $4.73+6.53*{10}^{-3}\Delta T-0.96*{10}^{-4}{\Delta T}^{2}$ | $8.38-8.05*{10}^{-2}\Delta T-0.10*{10}^{-5}{\Delta T}^{2}$ | = | ↑ |
|  | 3-4 |  | $6.59+7.97*{10}^{-5}\Delta T-0.70*{10}^{-5}{\Delta T}^{2}$ | $5.62+1.58*{10}^{-2}\Delta T+0.20*{10}^{-3}{\Delta T}^{2}$ | $9.94-7.72*{10}^{-3}\Delta T+0.39*{10}^{-3}{\Delta T}^{2}$ | ↓ | = |

Figure S 1: Spectra of N-acetylaspartate (NAA) referenced to DSS (pH 7, 40°C) (A) and example spectra for the pH dependence of the resonances in the upfield region (B) and the temperature dependence of the NH resonance (C). Experimentally determined ^1^H chemical shifts (δ [ppm]) and J-coupling constants (J[Hz]) and the fitted model functions (δ(pH, T), J(pH, T)) for the signals of NAA (D-K).


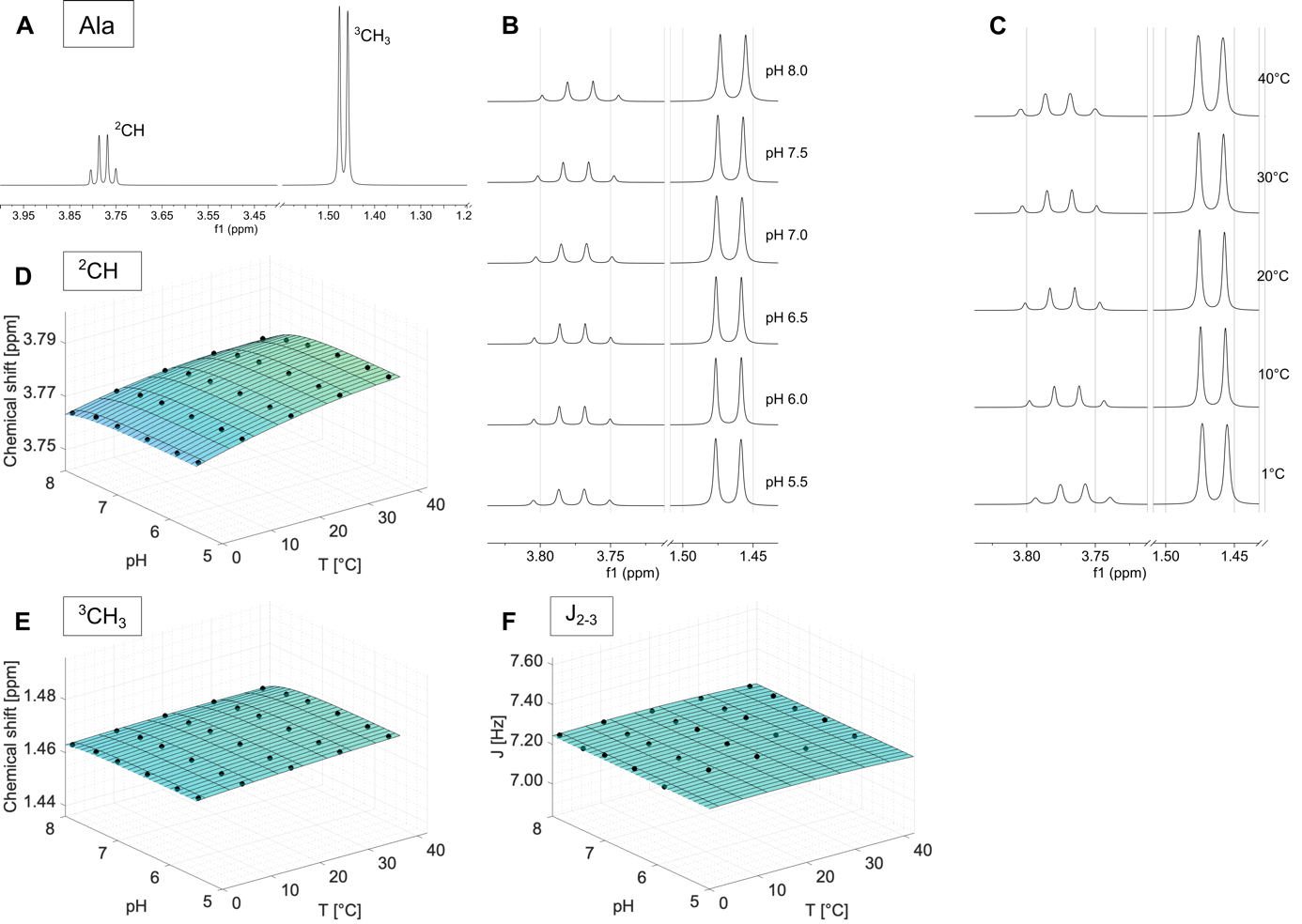


Figure S 2: Spectra of alanine (Ala) referenced to DSS (pH 7, 40°C) (A) and example spectra for the pH (B) and the temperature (C) dependence of the resonances. Experimentally determined ^1^H chemical shifts (δ [ppm]) and J-coupling constants (J[Hz]) and the fitted model functions (δ(pH, T), J(pH, T)) for the signals of Ala (D-F).

Figure S 3: Spectra of γ-Aminobutyric acid (GABA) referenced to DSS (pH 7, 40°C) (A) and example spectra for the temperature dependence of the resonances (B). Experimentally determined ^1^H chemical shifts (δ [ppm]) and J-coupling constants (J[Hz]) and the fitted model functions (δ(pH, T), J(pH, T)) for the signals of GABA (C-F).


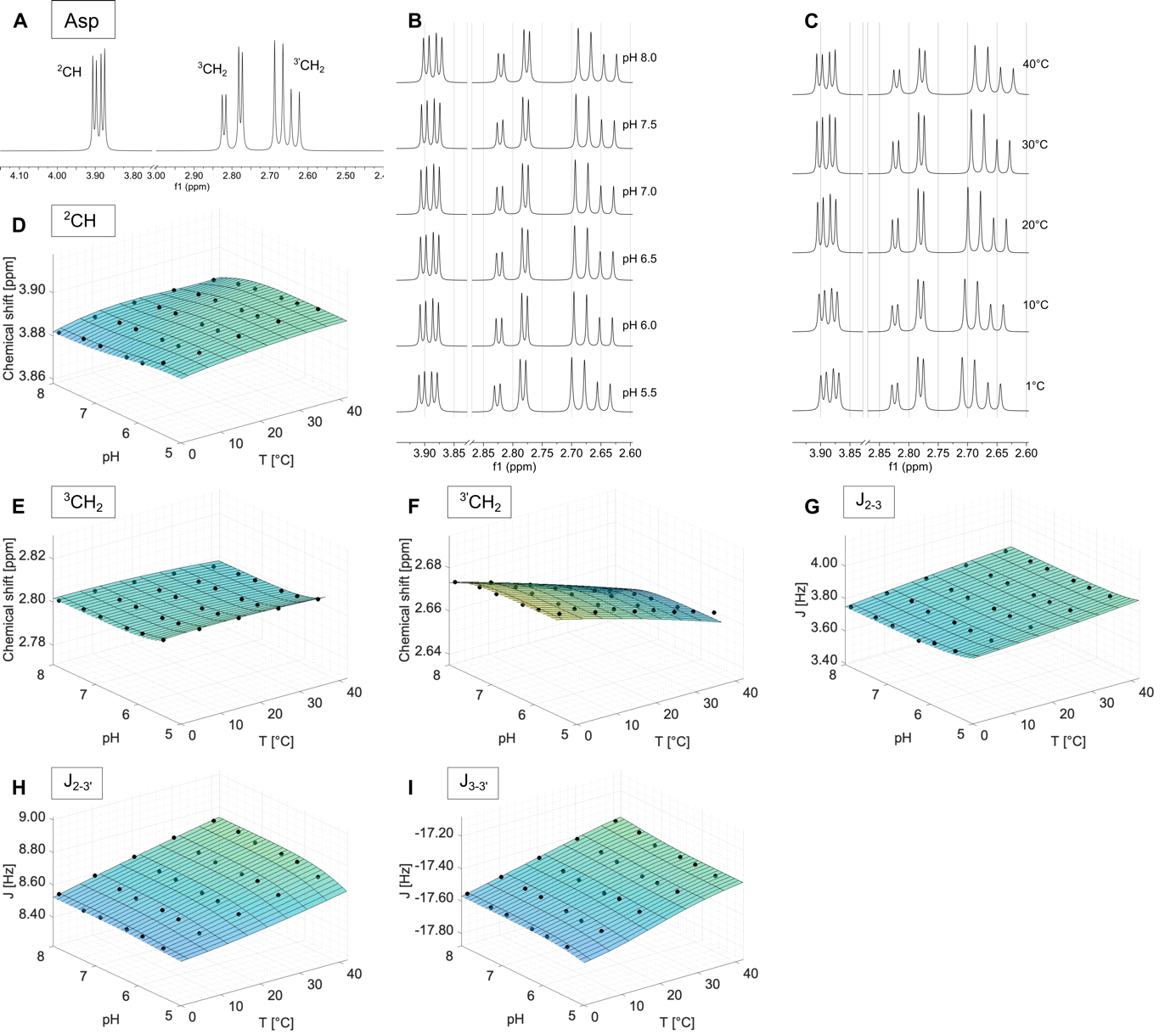


Figure S 4: Spectra of aspartate (Asp) referenced to DSS (pH 7, 40°C) (A) and example spectra for the pH (B) and temperature (C) dependence of the resonances. Experimentally determined ^1^H chemical shifts (δ [ppm]) and J-coupling constants (J[Hz]) and the fitted model functions (δ(pH, T), J(pH, T)) for the signals of Asp (D-I).

Figure S 5: Spectra of choline (Cho) referenced to DSS (pH 7, 40°C) (A) and example spectra for the temperature dependence of the resonances (B). Experimentally determined ^1^H chemical shifts (δ [ppm]) and the fitted model functions (δ(pH, T)) for the signals of Cho (C-E).

Figure S 6: Spectra of creatine (Cr) referenced to DSS (pH 7, 40°C) (A) and example spectra for the temperature dependence of the resonances (B) and the pH dependence of the NH resonance (C). Experimentally determined ^1^H chemical shifts (δ [ppm]) and the fitted model functions (δ(pH, T)) for the signals of Cr (D-F).


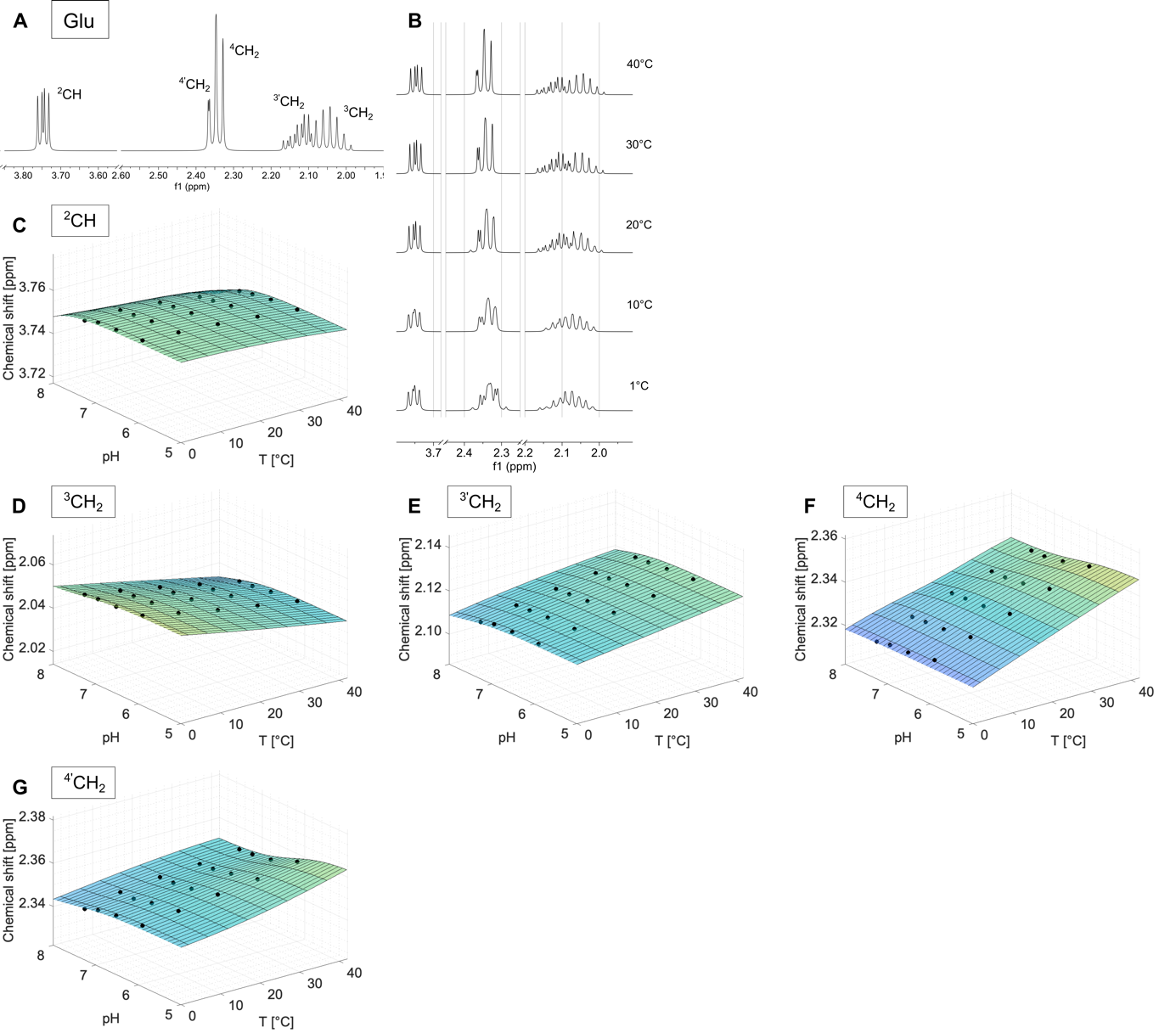


Figure S 7: Spectra of glutamate (Glu) referenced to DSS (pH 7, 40°C) (A) and example spectra for the temperature dependence of the resonances (B). Experimentally determined ^1^H chemical shifts (δ [ppm]) and the fitted model functions (δ(pH, T) for the signals of Glu (C-G).


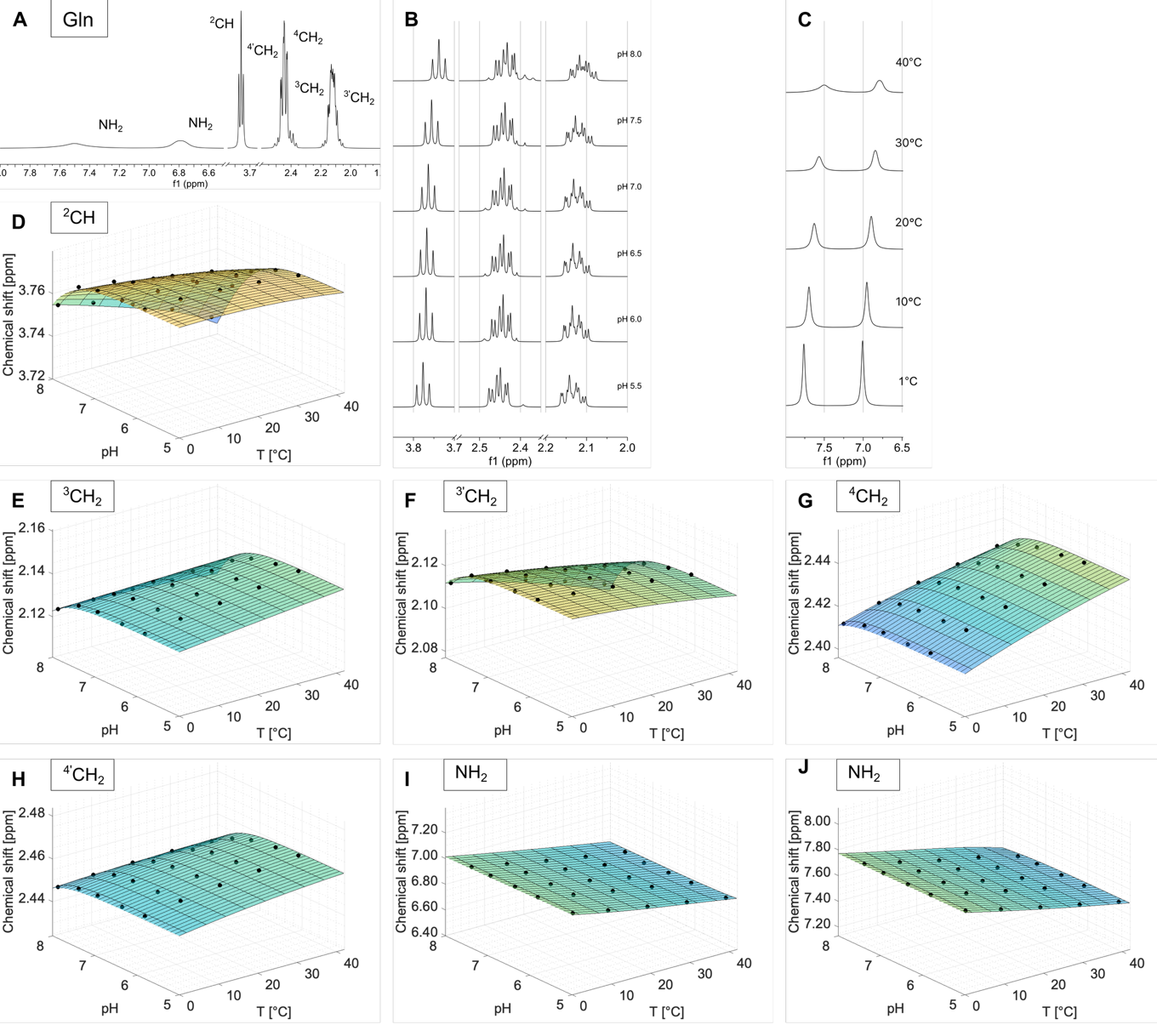


Figure S 8: Spectra of glutamine (Gln) referenced to DSS (pH 7, 40°C) (A) and example spectra for the pH dependence of the resonances in the upfield region (B) and the temperature dependence of the NH resonances (C). Experimentally determined ^1^H chemical shifts (δ [ppm]) and the fitted model functions (δ(pH, T) for the signals of Gln (D-J).

Figure S 9: Spectra of glycine (Gly) referenced to DSS (pH 7, 40°C) (A) and example spectra for the temperature dependence of the singlet (B). Experimentally determined ^1^H chemical shifts (δ [ppm]) and the fitted model function (δ(pH, T) for the singlet of Gly (C).


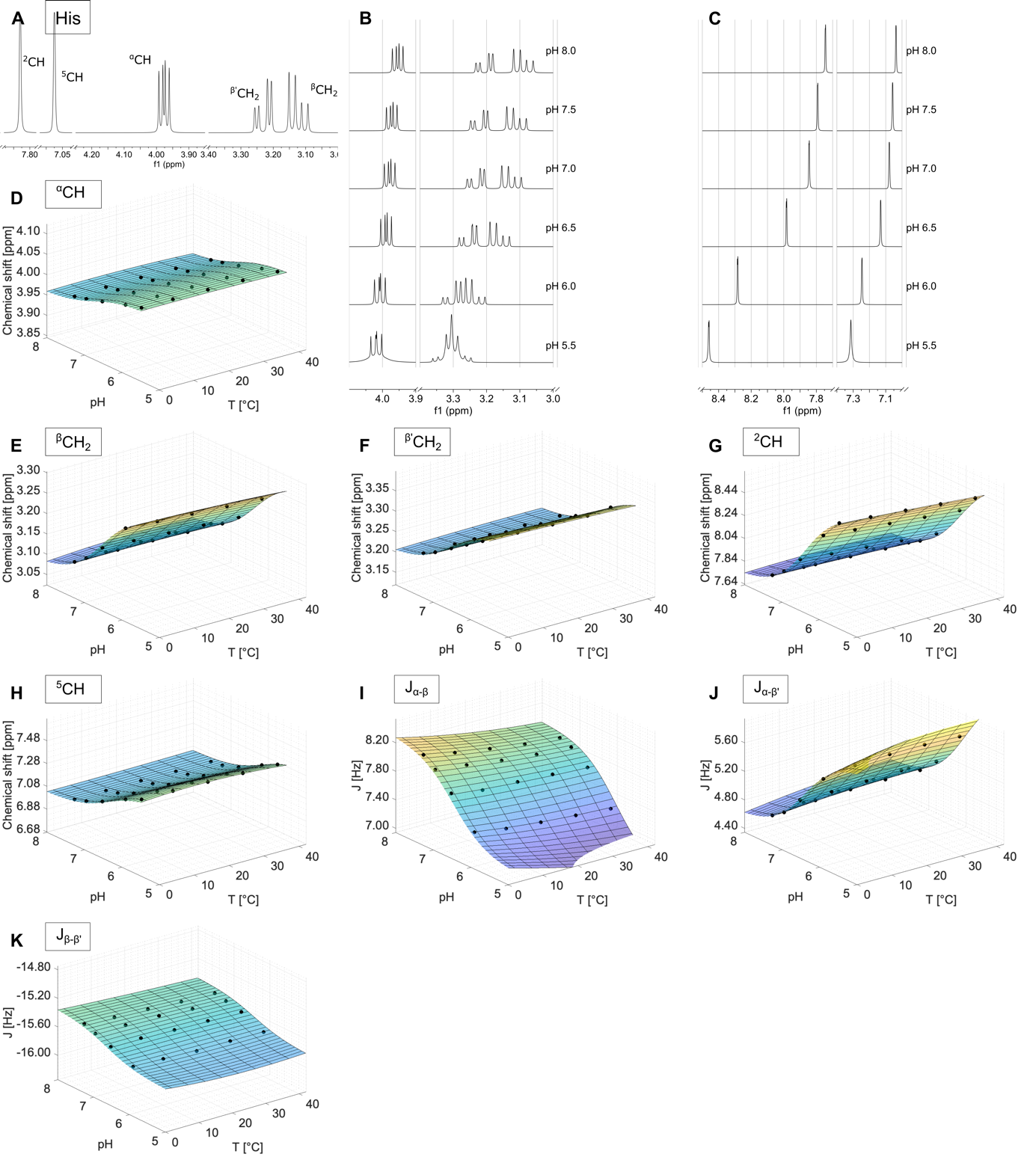


Figure S 10: Spectra of histidine (His) referenced to DSS (pH 7, 40°C) (A) and example spectra for the pH dependence of the resonances in the upfield region (B) and of the NH resonances (C). Experimentally determined ^1^H chemical shifts (δ [ppm]) and J-coupling constants (J[Hz]) and the fitted model functions (δ(pH, T), J(pH, T)) for the signals of His (D-K).

Figure S 11: Spectra of myo-inositol (m-Ins) referenced to DSS (pH 7, 40°C) (A) and example spectra for the pH dependence of the resonances (B). Experimentally determined ^1^H chemical shifts (δ [ppm]) and the fitted model functions (δ(pH, T)) for the signals of m-Ins (C-F).

Figure S 12: Spectra of lactate (Lac) referenced to DSS (pH 7, 40°C) (A) and example spectra for the temperature dependence of the resonances (B). Experimentally determined ^1^H chemical shifts (δ [ppm]) and J-coupling constant (J[Hz]) and the fitted model functions (δ(pH, T), J(pH, T)) for the signals of Lac (C-E).

Figure S 13: Spectra of phosphocreatine (PCr) referenced to DSS (pH 7, 40°C) (A) and example spectra for the temperature dependence of the resonances in the upfield region (B) and of the NH resonances (C). Experimentally determined ^1^H chemical shifts (δ [ppm]) and the fitted model functions (δ(pH, T)) for the signals of PCr (D-G).

Figure S 14: Spectra of taurine (Tau) referenced to DSS (pH 7, 40°C) (A) and example spectra for the pH dependence of the resonances (B). Experimentally determined ^1^H chemical shifts (δ [ppm]) and the fitted model functions (δ(pH, T) for the signals of Tau (C-D).

Figure S 15: Spectra of threonine (Thr) referenced to DSS (pH 7, 40°C) (A) and example spectra for the pH dependence of the resonances. Experimentally determined ^1^H chemical shifts (δ [ppm]) and J-coupling constants (J[Hz]) and the fitted model functions (δ(pH, T), J(pH, T)) for the signals of Thr (C-G).





Figure S 16: Metabolite concentrations determined by the AQSES algorithm for the examined temperature and pH range. The results are given as a percentage of the simulated values.


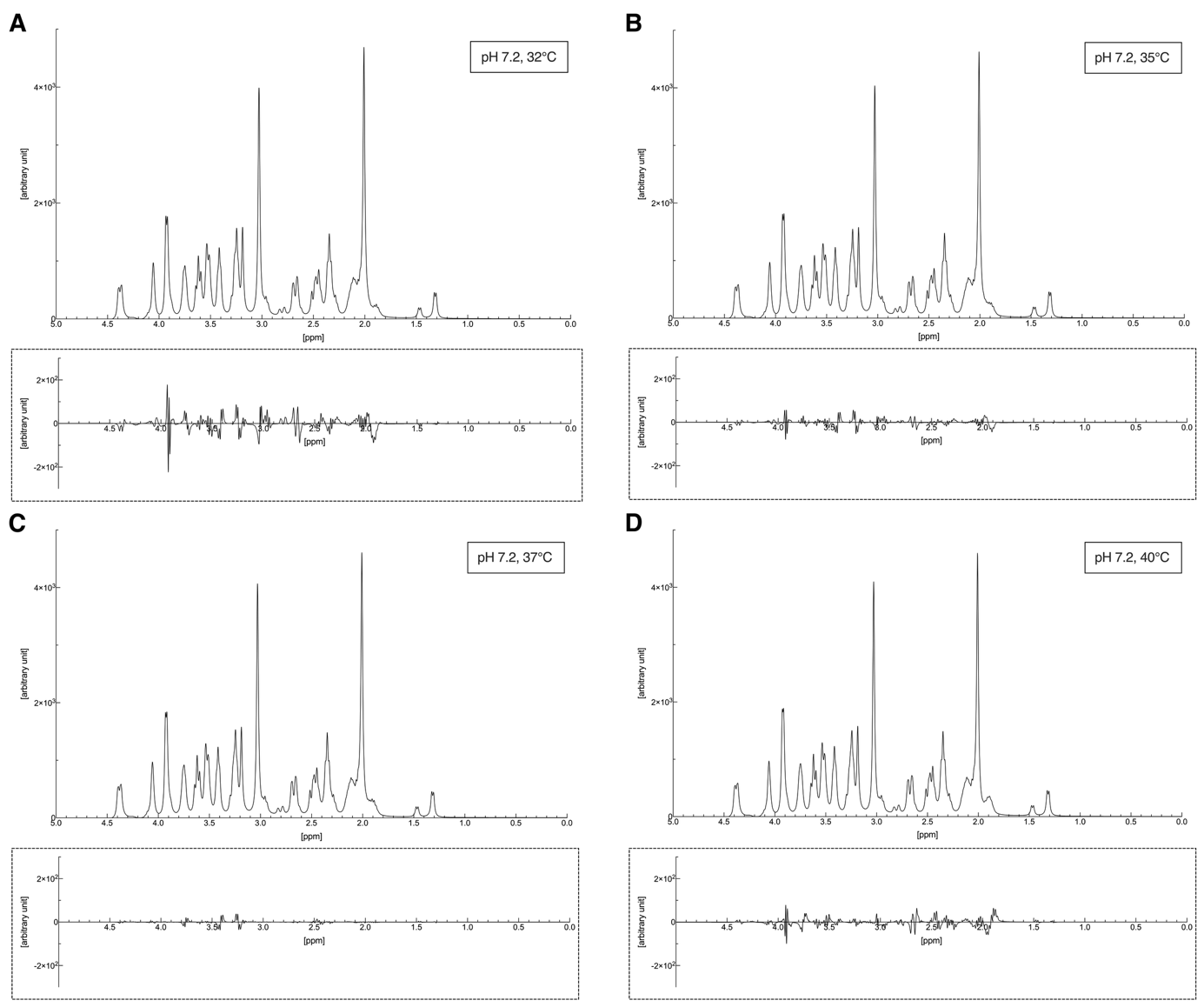


Figure S 17: ^1^H NMR spectrum simulated for pH 7.2 and 32°C, and the resulting residual spectrum using the AQSES algorithm, with chemical shift values and J-coupling constants for 37°C and pH 7 as prior knowledge (A). Subplots (B), (C), and (D) show the spectra for (pH 7.2 and 35°C, 37°C and 40°C), respectively, and also the corresponding residual spectra.


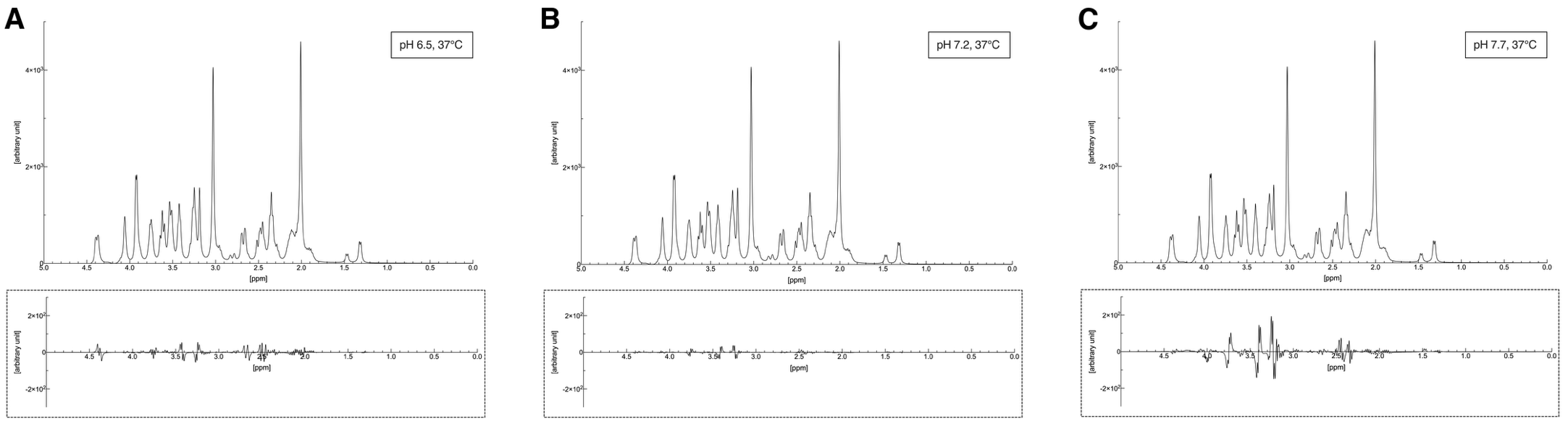


Figure S 18: ^1^H NMR spectrum simulated for pH 6.5 and 37°C, and the resulting residual spectrum using the AQSES algorithm, with chemical shift values and J-coupling constants for 37°C and pH 7 as prior knowledge (A). Subplots (B), and (C) show the spectra for (pH 7.2 and pH 7.7, 37°C), respectively, and also the corresponding residual spectra.
